# African Swine Fever Virus Structural Protein p17 Inhibits cGAS-STING Signaling Pathway Through Interacting With STING

**DOI:** 10.3389/fimmu.2022.941579

**Published:** 2022-07-01

**Authors:** Wanglong Zheng, Nengwen Xia, Jiajia Zhang, Qi Cao, Sen Jiang, Jia Luo, Hui Wang, Nanhua Chen, Quan Zhang, François Meurens, Jianzhong Zhu

**Affiliations:** ^1^ College Veterinary Medicine, Yangzhou University, Yangzhou, China; ^2^ Joint International Research Laboratory of Agriculture and Agri-Product Safety, Yangzhou University, Yangzhou, China; ^3^ Comparative Medicine Research Institute, Yangzhou University, Yangzhou, China; ^4^ Jiangsu Co-Innovation Center for Prevention and Control of Important Animal Infectious Diseases and Zoonoses, Yangzhou University, Yangzhou, China; ^5^ BIOEPAR, INRAE, Oniris, Nantes, France; ^6^ Department of Veterinary Microbiology and Immunology, Western College of Veterinary Medicine, University of Saskatchewan, Saskatoon, SK, Canada

**Keywords:** African swine fever virus, p17 protein, cGAS-STING pathway, TBK1, IKKϵ

## Abstract

African swine fever virus (ASFV) encodes more than 150 proteins, which establish complex interactions with the host for the benefit of the virus in order to evade the host’s defenses. However, currently, there is still a lack of information regarding the roles of the viral proteins in host cells. Here, our data demonstrated that ASFV structural protein p17 exerts a negative regulatory effect on cGAS-STING signaling pathway and the STING signaling dependent anti-HSV1 and anti-VSV functions. Further, the results indicated that ASFV p17 was located in ER and Golgi apparatus, and interacted with STING. ASFV p17 could interfere the STING to recruit TBK1 and IKKϵ through its interaction with STING. It was also suggested that the transmembrane domain (amino acids 39–59) of p17 is required for interacting with STING and inhibiting cGAS-STING pathway. Additionally, with the p17 specific siRNA, the ASFV induced IFN-β, ISG15, ISG56, IL-6 and IL-8 gene transcriptions were upregulated in ASFV infected primary porcine alveolar macrophages (PAMs). Taken together, ASFV p17 can inhibit the cGAS-STING pathway through its interaction with STING and interference of the recruitment of TBK1 and IKKϵ. Our work establishes the role of p17 in the immune evasion and thus provides insights on ASFV pathogenesis.

## Introduction

African swine fever virus (ASFV), the causative agent of African swine fever (ASF) which is a contagious disease of domestic pigs and wild boar, has spread globally in recent years with serious economic consequences for the swine industry. ASFV contains a linear double-stranded DNA genome that varies in length from about 170 to 193 kb and encodes 150-200 viral proteins, including 68 structural proteins and more than 100 non-structural proteins (1). Monocyte–macrophages are the main target cells of ASFV (2). The viruses are identified and attacked by the host immune system. In order to replicate and spread, ASFV has evolved multiple strategies to escape the host’s defense system (3). Several studies have suggested that ASFV could evade the innate immunity through the modulation of a number of host cell pathways controlling type I interferon (IFN-I) production and signaling (4), host cell general gene transcription and protein synthesis (5), cell proliferation and death (6, 7), and inflammatory response more specifically (3).

Accumulating evidences have demonstrated that inhibiting the production and the effects of IFN plays a critical role in ASFV pathogenesis (8). Several researches have indicated that virulent strains of ASFV, including Armenia/07, Lisboa60 and 22653/14 strains, have developed various measures to block the IFN production and responses in infected cells (9–11). Specifically, the virulent Armenia/07 strain was able to efficiently block the synthesis and the production of IFN-β mRNA in infected macrophages by inhibiting the cGAS-STING pathway (9). The virulent 22653/14 strain seemed to have developed mechanisms to suppress the induction of several type I IFN genes (10).The highly virulent strain Lisboa60 could inhibit IFN production in macrophages using a way independent on IRF3 modulation (11). Additionally, multiple ASFV proteins were shown to contribute to the inhibition of type I IFN responses by targeting various innate immune pathways, such as the cGAS-STING pathway, Toll-like receptors pathway, JAK/STAT pathway and NF-κB pathway (12–15).

P17 encoded by the ASFV D117L gene is located in the 140-150 kb of genome central region and close to the right variable region. P17 is a major structural transmembrane protein localized in the capsid and inner lipid envelope, and it is highly abundant and essential for the assembly and maturation of the icosahedral capsid as well as general virus viability (16). Several studies have indicated that the outer capsid shell was formed by the major capsid protein (p72) and four stabilizing minor proteins (H240R, M1249L, p17, p49) (17, 18). The p17 protein appears to form trimers and is located at the interface of the center gap region of three neighboring pseudo-hexameric capsomers (17). P17 closely associates with the base domain of p72, and three copies of p17 encircle each p72 trimer capsomer in the inner capsid shell, firmly anchoring p72 capsomers on the inner membrane (18). Our previous study also indicated that p17 protein could inhibit cell proliferation by inducing cell cycle arrest *via* ER stress-ROS pathway (19).

It is known that mammalian innate immune system utilizes pattern recognition receptors (PRRs) to detect pathogen-associated molecular patterns (PAMPs) from invading pathogens and activate the host innate immune response (20). Upon DNA virus infection, viral DNA is detected by cytosolic sensors. The cytosolic cyclic GMP-AMP (cGAMP) synthase (cGAS) plays a key role in sensing cytosolic DNA and triggering the stimulator of interferon genes (STING) dependent signaling to induce type I IFNs (21). cGAS is activated upon binding with double-stranded DNA and then catalyzes the second messenger 2’3’-cGAMP generation, which in turn activates STING (22). Activated STING is translocated from endoplasmic reticulum (ER) *via* ER Golgi intermediate compartment (ERGIC) to the Golgi apparatus and serves as a platform for recruitment and phosphorylation of TBK1 and interferon regulatory factor 3 (IRF3) (23). Upon recruitment and oligomerization, TBK1 phosphorylates itself, then phosphorylates STING and transcription factor IRF3 sequentially. Phosphorylated IRF3 dimerizes and translocates to the nucleus, where it triggers the production of type I IFNs leading to the expressions of interferon-stimulated genes (ISGs), which orchestrate antiviral defense mechanisms (22).

Previous studies have reported that virulent strains of ASFV could alter the production of IFN-β through the inhibition of the cGAS-STING pathway, and several proteins encoded by ASFV could inhibit cGAS-STING pathway through different mechanisms (9, 24). However, the molecular mechanism of ASFV modulating the cGAS-STING pathway has not been fully elucidated. Thus, we have analyzed the effects of whole genomic open reading frames (ORFs) from ASFV China 2018/1 on the activation of cGAS-STING pathway (25), and found that p17 was able to inhibit the porcine cGAS-STING mediated IFN signaling. Our work pointed the role of p17 in the immune evasion and help understand the ASFV pathogenesis.

## Materials and Methods

### Antibodies and Reagents

The rabbit TBK1 mAb (3504S), phosphorylated-TBK1 mAb (p-TBK1, 5483S), IRF3 mAb (11904S), FLAG mAb (14793), HA mAb (3724) and GFP mAb (2956) were acquired from Cell Signaling Technology (Boston, MA, USA). The rabbit p-IRF3 (Ser385), pAb(MA5-14947) was purchased from Thermo Fisher (Sunnyvale, CA, USA). The rabbit STING pAb (19851-1-AP) and Myc pAb (16286-1-AP) were purchased from ProteinTech (Wuhan, Hubei, China). The dsRed mAb (ab185921) was acquired from Abcam (Cambridge, Cambridgeshire, UK). The mouse cGAS mAb (sc-515777) was obtained from Santa Cruz Biotechnology (Santa Cruz, CA, USA). HRP goat anti-rabbit IgG (H+L) highly cross-adsorbed secondary antibody and goat anti-mouse IgG (H+L) highly cross-adsorbed secondary antibody, goat anti-rabbit IgG (H+L) highly cross-adsorbed secondary antibody Alexa Fluor Plus 488 (A32731) or Alexa Fluor Plus 647 (A32733), and goat anti-mouse IgG (H+L) highly cross-adsorbed secondary antibody Alexa Fluor 488 (A11029) were all acquired from Thermo Fisher (Sunnyvale, CA, USA). Mouse anti-ASFV p30 mAb was nicely provided by Drs. Xinyu Zhang and Xilong Kang in Yangzhou University.

DMEM and RPMI 1640 media were obtained from Hyclone (Hyclone Laboratories, Logan, Utah, USA). Fetal bovine serum (FBS) was obtained from Gibco (Grand Island, NY, USA). Double-luciferase reporter assay kit was bought from TransGen Biotech (Beijing, China). 2’3’-cGAMP and polydA:dT were bought from InvivoGen (Hong Kong, China) and used as the agonists for STING and cGAS, respectively. Lipofectamine 2000 was obtained from Invitrogen (Carlsbad, CA, USA). Protein A/G PLUS-Agarose was bought from Santa Cruz Biotechnology (sc-2003, CA, USA). DAPI staining solution (C1005), Enhanced BCA protein assay kit (P0010S) was purchased from Beyotime (Shanghai, China).

### Cells, Cell Transfection and Viruses

Human embryonic kidney (293T) cells (ATCC Cat No: CRL-3216) were maintained in a DMEM medium supplemented with 10% fetal bovine serum, 1 mM glutamine, and 1% penicillin/streptomycin, and maintained at 37°C with 5% CO_2_. Porcine pulmonary alveolar macrophages cell lines 3D4/21 (ATCC Cat No: CRL-2843) and primary porcine pulmonary alveolar macrophages (PAMs) were maintained in an RPMI 1640 medium supplemented with 10% fetal bovine serum, 1 mM glutamine, and 1% penicillin/streptomycin, and maintained at 37°C with 5% CO_2_. Monkey kidney epithelial cells (Vero, ATCC Cat No: CRL-1586) were maintained in a DMEM medium supplemented with 10% fetal bovine serum, 1 mM glutamine, and 1% penicillin/streptomycin, and maintained at 37°C with 5% CO_2_. Transfection was performed by using the Lipofectamine 2000 following the manufacturer’s instructions. The Vesicular Stomatitis Virus (VSV-GFP) and Herpes Simplex Virus-1 (HSV-1-GFP) were both provided by Dr. Tony Wang in SRI International USA, and used as described previously (26).

### Plasmids and Molecular Cloning

The D117L (p17) gene of ASFV China 2018/1 (GenBank submission No. MH766894) was synthesized and cloned into the *Hind* III and *Kpn* I sites of 3 x FLAG-CMV-7.1 vector. Next, the p17 gene was PCR amplified from pCMV-3FLAG-p17 and then cloned into the *EcoR* I and *EcoR* V sites of pCAGGS-2HA vector and *Bgl* II and *EcoR* I sites of pDsRed-Express-C1 vector, using the MultiF Seamless Assembly Mix (ABclonal, Wuhan China). The recombinant plasmids were named pCAGGS-p17-2HA and pDsRed-p17, respectively. Porcine STING (pSTING) was amplified by PCR from pDEST47-pSTING-2HA plasmid we prepared before, and cloned into the *Bgl* II and *EcoR* I sites of pEGFP-C1 vector to obtain pEGFP-pSTING. The PCR primers of p17 and pSTING are available in **Supplementary Table 1**.

Porcine cGAS, IFI16, IKK, IRF3 and p65 were cloned in either pcDNA-2HA or pEGFP-C1 and used as we described before (27). Human TBK1 (pcDNA-Myc-TBK1) and IKK (pcDNA-Myc-IKK) were cloned and preserved in our lab.

### Promoter-Driven Luciferase Reporter Gene Assay

Cells grown in 96-well plates (3×10^4^ cells/well) were co-transfected using Lipofectamine 2000 with IFN-β firefly luciferase, ISRE firefly luciferase or NF-κB firefly luciferase reporters (10 ng/well) (Fluc) and β-actin *Renilla* luciferase (Rluc) reporter (0.2 ng/well), together with the indicated plasmids or vector controls (5–40 ng/well). The total DNA per well was normalized to 50 ng by adding empty vector. After 24 hours (h) post- transfection, cells were harvested and lysed using lysis buffer for 15 min at room temperature (RT). Relative luciferase activity was measured using a Double-luciferase reporter assay kit following the manufacturer’s suggestions. The relative luciferase activity was analyzed by normalizing Fluc to Rluc activity. The results were expressed as fold induction of IFN-β-Fluc, ISRE-Fluc or NF-κB-Fluc compared with vector control group after Fluc normalization by corresponding Rluc.

### RNA Extraction and RT-qPCR

Total RNA was extracted by using TRIpure reagent following the manufacturer’s suggestions. The extracted RNA was reverse transcribed into cDNA using HiScript 1^st^ strand cDNA synthesis kit (Vazyme, Nanjing, China), and then the target gene expressions were measured using quantitative PCR (qPCR) with SYBR qPCR master Mix (vazyme, Nanjing, China) in StepOnePlus equipment (Applied Biosystems). The qPCR program is denaturation at 94°C for 30 seconds (s) followed by 40 cycles of 94°C for 5 s and 60°C for 30 s. All the qPCR assay efficiencies were between 90-100%. The relative mRNA levels were normalized to β-actin mRNA levels, and calculated using 2^−ΔΔCT^ method. The sequence of qPCR primers used are shown in **Supplementary Table 2**.

### Western Blotting and Co-Immunoprecipitation Analysis

Whole cell proteins were extracted with an RIPA lysis buffer. Then, the concentration of the whole protein was analyzed and adjusted using the BCA protein assay kit. The protein samples were mixed with 4×SDS sample buffer and boiled for 10 minutes (min). The protein supernatants were run by SDS-PAGE, and then the proteins in gel were transferred to PVDF membranes. The membranes were incubated with 5% skim milk solution at RT for 2 h, probed with the indicated primary antibodies at 4°C overnight, washed, and then incubated with secondary antibodies for 1 h at RT. The protein signals were detected by ECL detection substrate and imaging system.

For co-immunoprecipitation (Co-IP), the cleared cell lysate from transfected cells in 6-well plate (6-8×10^5^ cells/well) was incubated with 1 μg specific antibodies at 4°C overnight with shaking and further incubated with Protein A/G PLUS-Agarose for 2-3 h. The agarose was washed and eluted with 40 μL of 2 × SDS sample buffer. The elution samples together with input controls in 1×SDS sample buffer were both subjected to Western blotting.

### Immunofluorescence and Confocal Microscopy

Cells cultured over cell coverslips in a 24-well plate (1-2×10^5^ cells/well) were fixed in 4% paraformaldehyde for 30 min at RT, permeabilized by 0.5% Triton X-100, and then blocked with 5% BSA. The treated cells were incubated with primary antibody against FLAG, HA or TBK1 (1: 500) overnight, and then incubated with secondary antibody (1: 500) for 1 h. Finally, the coverslips were counterstained with DAPI, loaded on slide, sealed by nail polish, and visualized under a confocal laser-scanning microscope (Leica TCS SP8, Leica, Weztlar, Germany). Green fluorescence protein GFP and red fluorescence protein dsRed expressions in cells were directly visualized by confocal microscopy. The images were acquired at 63x magnification, and the size of each image is 1024 x 1024.

### Flow Cytometry

3D4/21 cells seeded in 12-well plates (3×10^5^ cells/well) were transfected with p17 plasmid (1 μg) for 24 h, and next stimulated by transfection with 2’3’-cGAMP (1 μg/mL) for 12 h. The cells were then infected with HSV1-GFP (MOI 0.01) for 20 h. 293T cells seeded in 12-well plates (3×10^5^ cells/well) were transfected with p17 (0.5 μg) together with cGAS (0.5 μg) and STING (0.5 μg) plasmids for 12 h, and the control groups were transfected with empty plasmid (pCAGGS-2HA), and then infected with VSV-GFP (MOI 0.001) for 8 h. After infection, cells were harvested and washed twice with PBS. The cell samples were filtered using a 200-mesh nylon filter, and cell suspensions analyzed by flow cytometry at a wavelength pair of 488/525 nm. A total of 10000 events were acquired in a flow cytometer.

### Measurement of Antiviral Activity by Plaque Assay

Vero cells were seeded into 12-well plates (3×10^5^ cells/well). After the cells were grown into monolayer, cells were infected by the tenfold serially diluted cell supernatants from HSV1 or VSV infected cells for 2 h. Then the infected cells were washed with PBS and overlaid with immobilizing medium (1:1 mixture of warmed 2×DMEM with 4% FBS and a stock solution of heated 1.6% low melting agarose). Plaque formation required about 4 days and 2 days for HSV1 and VSV, respectively. Upon completion, the immobilizing medium were discarded by tipping and cells were fixed and stained with crystal violet cell colony staining solution (0.05% w/v crystal violet, 1% formaldehyde, 1×PBS and 1% methanol) for 1 h at room temperature. After staining, cells were washed with tap water until clear plaques appeared. The plaques were counted and photos were taken.

### ASFV Infection and siRNA Treatment

Primary PAMs were isolated from the lung lavage fluid of 4-week-old healthy piglets and maintained in RPMI-1640 medium containing 10% FBS. The fifth passaged genotype II ASFV (GenBank accession number ON456300) in primary PAMs was used for subsequent siRNA knockdown experiments. The primary PAMs (1×10^6^ cells/well) were transfected with p17 siRNAs and control siRNA (100 nM each, GenePharma) using Lipofectamine 2000, and 24 h later the transfected cells were infected with 0.01 MOI ASFV for another 72 h. The cells were harvested for RT-qPCR and Western blot analysis, respectively. All ASFV infection experiments were performed in the animal biosafety level 3 (ABSL-3) of Yangzhou University approved by the Ministry of Agriculture and Rural Affairs (07140020201109-1). The animal experiment was in strict accordance with the Guidance for the Care and Use of Laboratory Animals of Yangzhou University (SYXK(JS)-2021-0026).

Additionally, the porcine STING siRNA were synthesized based on our previous study (28). One day before transfection, 3D4/21 cells were plated into 24-well plates and cultured in growth medium. The cells with 50% confluency were transfected with 50 nM STING siRNA and control siRNA using Lipofectamine 2000. All the siRNA sequences used are listed in in **Supplementary Table 3**.

### Statistical Analysis

All of the experiments were representative of three similar experiments. The results in bar graphs were analyzed using GraphPad Prism 6 software and presented as the mean ± standard deviation (SD) with three replicates. Statistical analysis was performed using Student’s *t*-test or *ANOVA* where appropriate; *p* < 0.05 was considered statistically significant. In the figures, “*”, “**” and “ns” denote *p* < 0.05, *p* < 0.01 and statistically not significant, respectively.

## Results

### ASFV p17 Can Inhibit the DNA Sensing Porcine cGAS-STING Signaling Pathway

**Figure 1:**
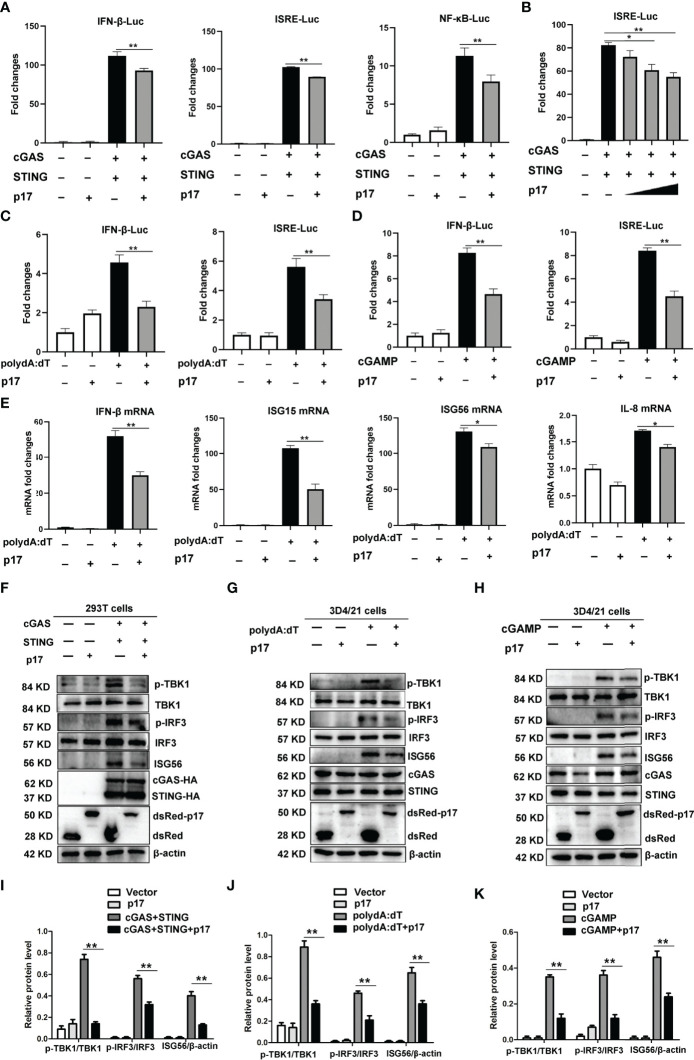
The effects of ASFV p17 on the porcine DNA sensing cGAS-STING pathway. **(A, B)** 293T cells grown in 96-well plate were transfected with pcGAS (20 ng), pSTING (10 ng), pdsRed-p17 (10 ng) plus IFN Fluc/ISRE Fluc/NF-κB Fluc and Rluc **(A)**, pcGAS (20 ng), pSTING (10 ng), pdsRed-p17 (5, 10, 20 ng) plus ISRE Fluc and Rluc **(B)**. **(C, D)** 3D4/21 cells grown in 96-well plate were transfected with pdsRed-p17 (20 ng), plus IFN-β-Fluc/ISRE-Fluc and Rluc for 12 h, and then stimulated by transfection of polydA:dT (1 μg/mL) **(C)** or 2’3’-cGAMP (2 μg/mL) **(D)** for another 12 h, followed by luciferase measurement. **(E)** 3D4/21 cells in 12-well plate were transfected with pdsRed-p17 (1 μg) for 12 h and then stimulated by transfection of polydA:dT (1 μg/mL) for another 12 h, followed by RT-qPCR detection of IFN-β, ISG15, ISG56 and IL-8 mRNA. **(F, I)** 293T cells in 12-well plate were transfected with pdsRed-p17 (0.5 μg), pcGAS (0.5 μg) and pSTING (0.5 μg) for 24 h **(G, J)** 3D4/21 cells in 12-well plate were transfected with pdsRed-p17 (1 μg), plus polydA:dT (1 μg/mL) for 24 h **(H, K)** 3D4/21 cells in 12-well plate were transfected with pdsRed-p17 (1 μg), plus 2’3’-cGAMP (2 μg/mL) for 24 h Relative protein values represent the mean ± S.D. *P < 0.05, **P < 0.01.

In order to explore the molecular mechanisms of immune evasion by ASFV, we analyzed the impact of p17 on the DNA sensing cGAS-STING pathway. Firstly, the effects of p17 on promoters’ activations, including IFN-β, ISRE and NF-κB, were analyzed using dual-luciferase reporter assay. The results from 293T cells showed that cGAS/STING stimulated activations of ISRE, IFN-β and NF-κB promoters were all decreased in the presence of p17 (**Figures 1A, B**). Similarly, in the p17 transfected 3D4/21 cells, the polydA:dT or 2’3’-cGAMP stimulated IFN-β and ISRE promoter activations were also decreased (**Figures 1C,D**). Secondly, the effects of p17 on the cGAS-STING pathway mediated downstream cellular mRNA expressions of IFN-β, ISG15, ISG56 and IL-8 were analyzed by RT-qPCR. It appeared that p17 could inhibit polydA:dT induced downstream mRNA expressions of IFN-β, ISG15, ISG56 and IL-8 (**Figure 1E**).

Furthermore, the effects of p17 on the cGAS-STING signaling pathway were examined using Western blotting in both 293T and 3D4/21 cells (**Figures 1F–K**). The exogenous porcine cGAS-STING induced phosphorylations of TBK1 (p-TBK1) and IRF3 (p-IRF3), and downstream ISG56 induction were all inhibited by p17 (**Figures 1F, I**). The polydA:dT and 2’3’-cGAMP both activated endogenous porcine cGAS-STING signaling, upregulating the levels of p-TBK1, p-IRF3 and downstream ISG56. With the expression of p17, the polydA:dT induced p-TBK1, p-IRF3 and downstream ISG56 were decreased (**Figures 1G, J**), and the 2’3’-cGAMP induced p-TBK1, p-IRF3 and downstream ISG56 were also decreased (**Figures 1H, K**).

**Figure 2:**
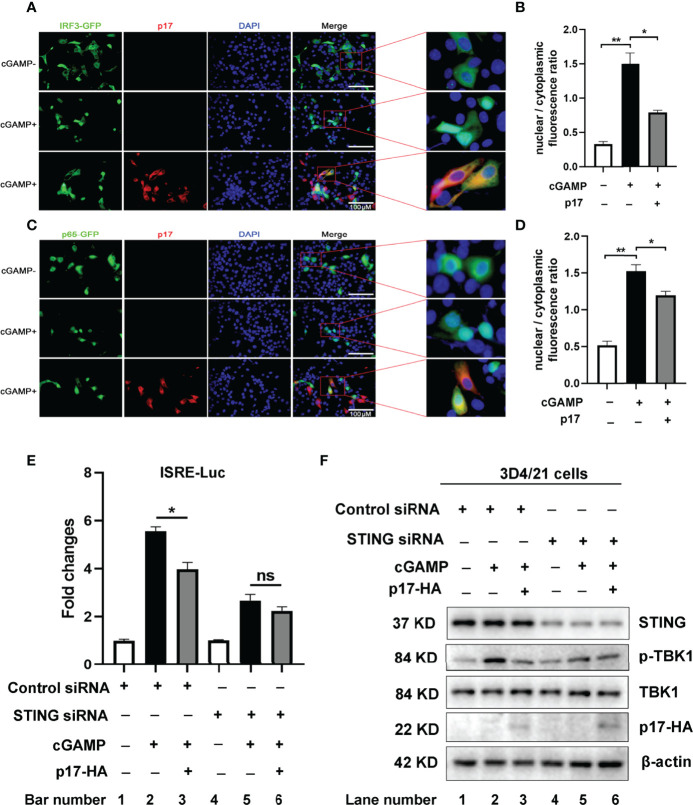
**(A-D)**The effects of ASFV p17 on nuclear translocations of IRF3 and NF-κB p65. The 3D4/21 cells grown on the coverslips in 24-well plates (1×10^5^ cells/well) were transfected with IRF3-GFP (0.5 μg) and pCAGGS-p17-2HA (0.5 μg) or control vector (0.5 μg) **(A, B)**, p65-GFP (0.5 μg) and pCAGGS-p17-2HA (0.5 μg) or control vector (0.5 μg) **(C, D)** using Lipofectamine 2000. At 18 h post translation, the cells were stimulated with transfection of 2’3’-cGAMP (1μg/ml) for another 6 (h) The cells on coverslips were sequentially probed with rabbit anti-HA mAb, goat anti-rabbit IgG H&L Alexa Fluor 594, counter-stained with nucleus marker DAPI. **(A)** The effects of ASFV p17 on nuclear translocations of IRF3 was observed by fluorescence microscope with the enlarged images shown on the right. **(B)** Statistical analysis of the ratios of IRF3-GFP in nuclear fractions vs cytoplasmic fractions. **(C)** The effects of ASFV p17 on nuclear translocations of p65 was observed by fluorescence microscope with the enlarged images shown on the right. **(D)** Statistical analysis of the ratios of p65-GFP in nuclear fractions vs cytoplasmic fractions. **(E, F)** 3D4/21 cells in 24-well plates were transfected with the STING siRNA or Control siRNA (50 nM) by lipofectamine 2000 for 24 h, followed by co-transfected p17, ISRE-Fluc and Rluc for 12 h, and then stimulated by cGAMP for another 12 h. **(E)** Effects of the activation of ISRE promoter were detected by luciferase measurement. **(F)** Effects of the phosphorylation of TBK1 (p-TBK1) were detected by Western blot. Fold change values represent the mean ± S.D. *P < 0.05, **P < 0.01, ns not significant.

Besides, we also checked the effect of p17 on the IRF3 and NF-κB p65 nuclear translocations. The results showed that in the presence of p17, the STING agonist 2’3’-cGAMP induced nuclear translocations of both IRF3 (**Figures 2A, B**) and p65 (**Figures 2C, D**) were inhibited. These results further consolidated the inhibitory effect of p17 on cGAS-STING signaling.

Additionally, we performed the experiments using porcine STING siRNA and control siRNA in 3D4/21 cells. The siRNA results in **Figures 2E, F** showed that in control siRNA treated cells, after stimulated by cGAMP, p17 significantly inhibits the activation of ISRE promoter (**Figure 2E** bar 3 versus bar 2), and the phosphorylation of TBK1 (p-TBK1) (**Figure 2F** lane 3 versus lane 2), whereas in STING siRNA treated cells, p17 does not further inhibit cGAMP stimulated the activation of ISRE promoter (**Figure 2E** bar 6 versus bar 5), and the phosphorylation of TBK1 (p-TBK1) (**Figure 2F** lane 6 versus lane 5). Taken together, these data suggest that ASFV p17 could inhibit the DNA sensing cGAS-STING signaling pathway.

### ASFV p17 Can Disturb the STING Signaling Mediated Antiviral Responses

**Figure 3:**
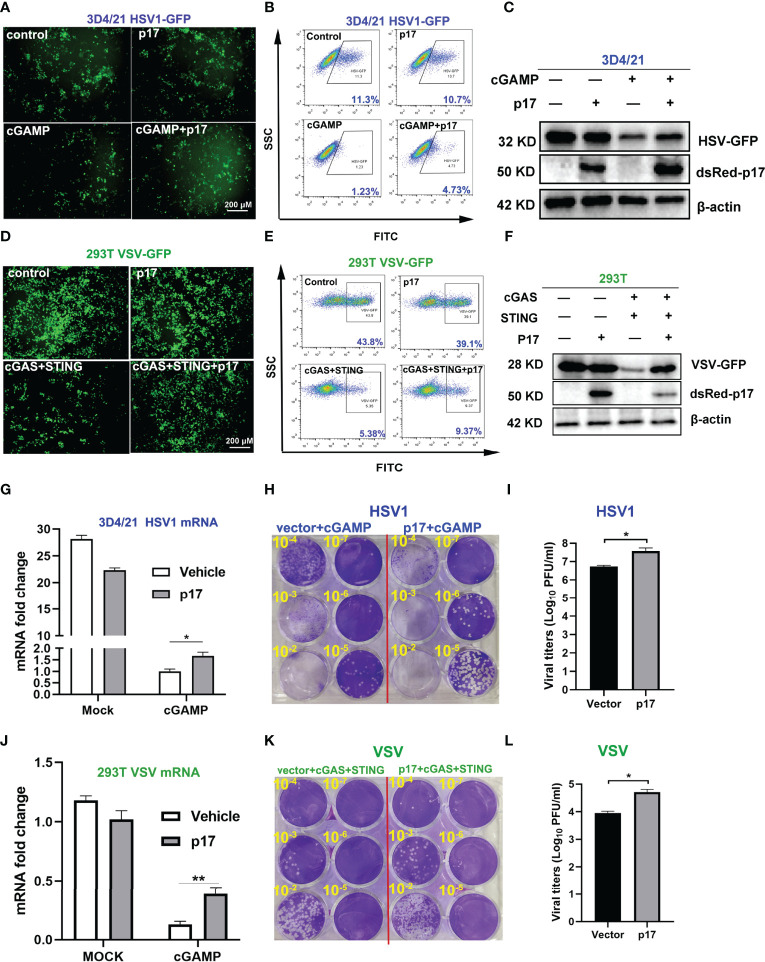
The effects of p17 on the cGAS-STING signaling mediated anti-HSV1 and anti-VSV activity. **(A-C, G-I)** 3D4/21 cells grown in 12-well plates (3×10^5^ cells/well) were transfected with pdsRed-p17 (1μg) for 12 h, and next stimulated by transfection with 2’3’-cGAMP (2 μg/mL) for 12 h. The cells were infected with HSV1-GFP at the MOI 0.01 for 20 h. **(A)** The HSV1 replicative GFP signals were observed by fluorescence microscopy. **(B)** The HSV1-GFP infected cells were analyzed by flow cytometry. **(C)** The viral GFP expressions were analyzed by Western blotting. **(G)** HSV1 gB gene expressions were analyzed by RT-qPCR. **(H)** The viral titers in the supernatants from HSV1 infected 3D4/21 cells were analyzed by plaque assay. **(I)** The plaque numbers were quantified and graphed. **(D-F, J-L)** 293T cells grown in 12-well plate (3×10^5^ cells/well) were co-transfected with pdsRed-p17 (0.5 μg), pcGAS (0.5 μg) and pSTING (0.5 μg) for 12 h, and then the cells were infected with VSV-GFP at the MOI 0.001 for 8 h. The VSV replicative GFP signals were observed by fluorescence microscopy **(D)**, analyzed by flow cytometry **(E)**, and by Western blotting **(F)**. The VSV glycoprotein genes were analyzed by RT-qPCR **(J)** and the VSV titers in supernatants from VSV infected 293T cells were measured by plaque assay **(K)**. The plaque numbers were quantified and graphed **(L)**. *P < 0.05, **P < 0.01.

In order to further investigate the impact of p17 on the cGAS-STING pathway, the effects of p17 on the STING signaling mediated antiviral responses were analyzed. 3D4/21 cells were infected with DNA virus HSV1-GFP, and STING agonist 2’3’-cGAMP showed obvious antiviral activity. However, ASFV p17 disturbed the 2’3’-cGAMP mediated anti-HSV1 activity, as evidenced by fluorescence microscopy (**Figure 3A**), flow cytometry (**Figure 3B**) and Western blotting (**Figure 3C**). Both the HSV1 gB gene expression assessed using RT-qPCR (**Figure 3G**) and virus titration determined by plaque assay (**Figures 3H, I**) showed similar results.

The cGAS-STING signaling pathway has been shown to have broad antiviral functions (26). Therefore, 293T cells were infected with RNA virus VSV-GFP, and co-expressed porcine cGAS and STING triggered obvious anti-VSV activity (**Figures 3D–F**). However, ASFV p17 disturbed anti-VSV activity mediated by co-expressing porcine cGAS and STING in 293T cells, which was evidenced by fluorescence microscopy (**Figure 3D**), flow cytometry (**Figure 3E**) and Western blotting (**Figure 3F**). Both the VSV glycoprotein gene expression assessed using RT-qPCR (**Figure 3J**) and virus titration determined by plaque assay (**Figures 3K, L**) showed similar results. Taken together, these data suggested that ASFV p17 could inhibit the cGAS-STING signaling mediated anti-HSV1 and anti-VSV responses.

### ASFV p17 Is Located in ER and Golgi Apparatus, and Co-Localized With STING Protein

**Figure 4:**
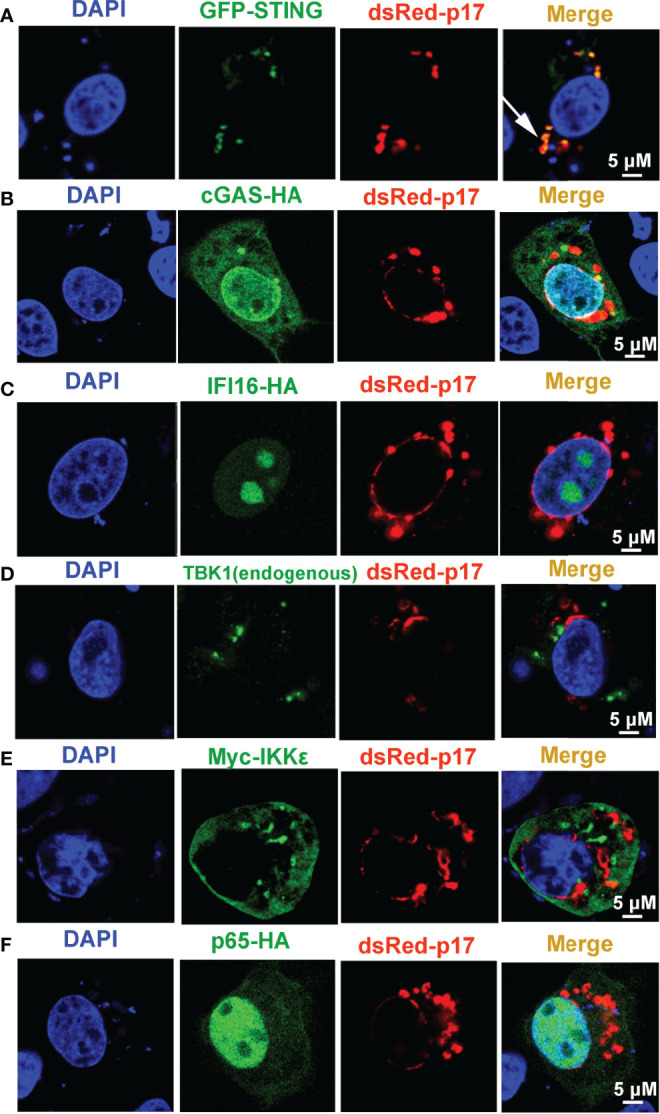
The cellular co-localizations between ASFV p17 and the related proteins of cGAS-STING pathway. 3D4/21 cells grown on glass coverslips in 24-well plate (1.5×10^5^ cells/well) were co-transfected with dsRed-p17 (0.5 μg) plasmids plus GFP-STING (0.5 μg) **(A)**, cGAS-HA (0.5 μg) **(B)**, IFI16-HA (0.5 μg) **(C)**, Myc-IKKϵ (0.5 μg) **(E)** and p65-HA (0.5 μg) **(F)**, respectively, as indicated. HA tagged proteins were stained with rabbit anti-HA antibody and Alexa Fluor plus 488 anti-rabbit second antibody. Myc-IKKϵ was stained with rabbit anti-Myc antibody and Alexa Fluor plus 488 anti-rabbit second antibody. Endogenous TBK1 was stained with rabbit anti-TBK1 antibody and Alexa Fluor plus 488 anti-rabbit second antibody **(D)**. GFP and dsRed expressions in cells were directly visualized by confocal microscopy. The cellular co-localizations of dsRed-p17 protein with various cGAS-STING pathway proteins in 3D4/21 cells was visualized by confocal fluorescence microscopy. The arrow illustrates the co-localized puncta pattern.

In order to investigate the molecular mechanism underlying the p17 inhibition of cGAS-STING pathway, the subcellular localization of p17 in cells, and the co-localization between p17 and signaling proteins of cGAS-STING pathway were assessed by immunofluorescence assay (IFA). The results from IFA showed that p17 protein was co-localized with the ER retention RFP marker (ER retention protein KDEL fused to RFP), and Golgi apparatus RFP marker (sialyltransferase fused to RFP), but not with the mitochondria-targeting GFP marker (mitochondrial targeting sequence of cytochrome c oxidase subunit VII fused to GFP) and lysosome LAMP1-GFP marker (lysosomal-associated membrane protein1 fused to GFP) (**Supplementary Figures 1A–E**). Furthermore, the IFA results also indicated that p17 was co-localized with STING protein, which is a ER resident protein, but not co-localization with cGAS, TBK1, IKKϵ, IFI16 and p65 (**Figures 4A–F**, **Supplementary Figure 3A**). Taken together, these data suggested that p17 is located in ER and Golgi apparatus, and co-localizes with STING protein.

### P17 Inhibits the cGAS-STING Pathway Through Its Interaction With STING and Its Interference in the Recruitment of TBK1 and IKKϵ

**Figure 5:**
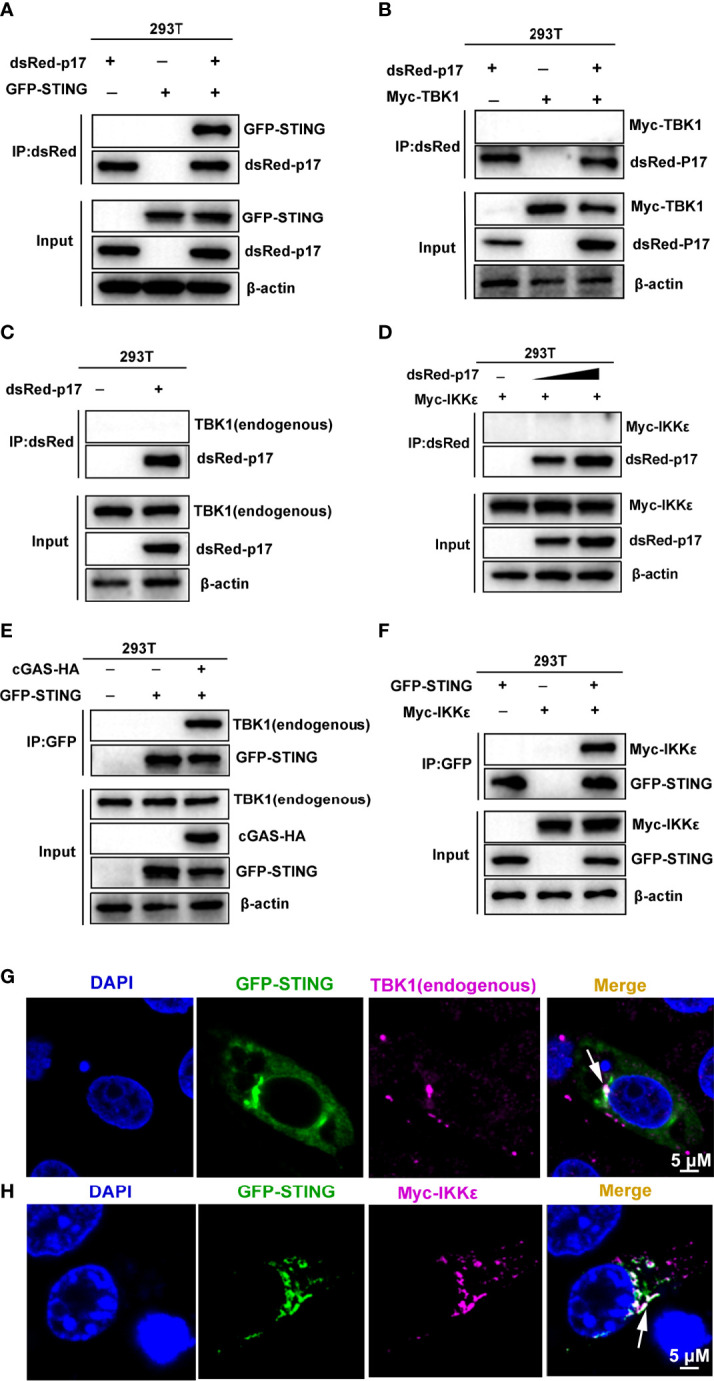
**(A–F)** Co-immunoprecipitation assays for analyzing the interactions between p17, STING, TBK1 and IKKϵ. **(A)** pDsRed-p17 (0.5 μg) and pGFP-STING (0.5 μg) were co-transfected into 293T cells in 6-well plate for 24 h **(B)** pDsRed-p17 (0.5μg) and pMyc-TBK1 (0.5 μg) were co-transfected into 293T cells for 24 h. **(C)** pDsRed-p17 (1 μg) was transfected into 293T cells for 24 h **(D)** pDsRed-p17 (0.5 μg and 1 μg) and pMyc-IKKϵ (0.5 μg) were co-transfected into 293T cells for 24 h. **(E)** The pGFP-STING (0.5μg) and pcGAS-HA (0.5 μg) were transfected into 293T cells for 24 h. **(F)** The pGFP-STING (0.5 μg) and pMyc-IKKϵ (0.5 μg) were co-transfected into 293T cells for 24 h The cell lysates were subjected for immuno-precipitation and subsequent Western blotting using the indicated tag antibodies. As a control in A, there is no interaction between GFP and dsRed (data not shown). **(G, H)** The cellular co-localizations between STING and TBK1/IKKϵ were analyzed by using IFA. **(G)** The pGFP-STING (1 μg) were transfected into 3D4/21 cells for 12h and then stimulated by transfection of polydA:dT (1 g/mL) for another 12h. The endogenous TBK1 was stained by rabbit anti-TBK1 mAb and Alexa Fluor plus 647 anti-rabbit second antibody, and the stained cells were examined by confocal microscopy. **(H)** The pGFP-STING (0.5 μg) and pMyc-IKKϵ (0.5 μg) were co-transfected into 3D4/21 cells for 24 h. The Myc-IKKϵ was stained with rabbit anti-Myc pAb and Alexa Fluor plus 647 anti-rabbit second antibody, and stained cells were observed by confocal microscopy. The arrows illustrate the co-localized areas.

Considering the cellular co-localization of ASFV p17 and STING, we hypothesized that ASFV p17 likely interferes with STING to affect the cGAS-STING pathway. Thus, we detected the interaction between p17 and STING protein using co-immunoprecipitation (Co-IP) and the results showed that p17 could interact with STING (**Figure 5A**), but it did not interact with either TBK1 or IKKϵ proteins (**Figures 5B–D**), suggesting the specific interaction between p17 and STING. To confirm and explore the roles of TBK1 and IKKϵ in the cGAS-STING signaling, the interactions between STING and these two signaling proteins were also examined by Co-IP. TBK1 was well-involved in STING signaling, and the results showed that upon STING activation, STING was able to interact with TBK1 as expected (**Figure 5E**). Regarding the role of IKKϵ in STING signaling, our results showed that STING can also interacted with it (**Figure 5F**). The data from IFA showed that STING co-localized with TBK1 and IKKϵ protein as punctate patterns confirming the Co-IP results (**Figures 5G, H**, and **Supplementary Figure 3B**).

**Figure 6:**
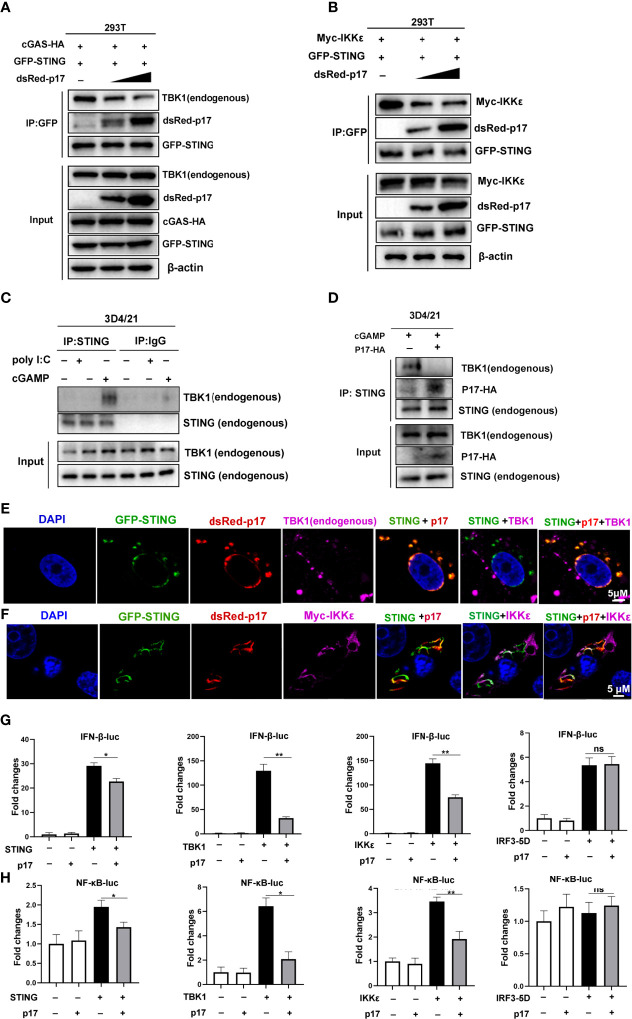
**(A, B)** The effects of p17 on the interactions and cellular co-localizations between STING and TBK1/IKKϵ. **(A)** 293T cells in 6-well plate were co-transfected with pcGAS-HA (0.5 μg) and pGFP-STING (0.5 μg) with or without pdsRed-p17 (0.5 μg and 1 μg) for 24 h **(B)** 293T cells were co-transfected with pGFP-STING (0.5 μg) and pMyc-IKKϵ (0.5 μg) with or without pdsRed-p17 (0.5 μg and 1 μg) for 24 **(h)** The cell lysates were subjected for immuno-precipitation and Western blotting using the indicated antibodies. **(C, D)** The effect of p17 on the interaction between endogenous STING and TBK1. **(C)** 3D4/21 cells were stimulated by transfection of poly I:C (1 μg/mL) or 2’3’-cGAMP (1 μg/mL), respectively, for 12h. The cell lysates were subjected for immune-precipitation with rabbit anti-STING pAb, and subsequent Western blotting using the rabbit anti-TBK1 mAb and anti-STING pAb. **(D)** The pCAGGS-p17-HA (2 μg) were transfected into 3D4/21 cells for 12h and then stimulated by transfection of 2’3’-cGAMP (1 μg/mL) for another 12h. The cell lysates were subjected for immune-precipitation and subsequent Western blotting. **(E, F)** The effect of p17 on the cellular co-localization between STING and TBK1/IKKε was analyzed by IFA. GFP and dsRed expression in cells was directly visualized by confocal microscopy. The endogenous TBK1 and Myc-IKK were stained by rabbit anti-TBK1 mAb and anti-Myc, respectively, followed by Alexa Fluor plus 647 anti-rabbit secondary antibody. **(G, H)** The effects of p17 on the activations of IFN promoter **(G)** and NF-κB promoter **(H)** triggered by transfected STING (20 ng), TBK1 (20 ng), IKKϵ (20 ng) and IRF3-5D (20 ng). The fold changes are relative to vector transfected controls. Values represent the mean ± S.D. *P < 0.05, **P < 0.01, ns not significant.

Based on above results, we explored the effects of ASFV p17 on the interaction between STING and TBK1/IKKϵ, Co-IP results showed that p17 was able to inhibit not only the interaction of STING and TBK1 (**Figure 6A**), but also the interaction of STING and IKKϵ (**Figure 6B**). Importantly, p17 could also inhibit endogenous interaction of STING and TBK1 (**Figures 6C, D**). The IFA assay also suggested that in the presence of p17, the co-localizations between STING and TBK1, and between STING and IKKϵ disappeared (**Figures 6E, F**, and **Supplementary Figure 3C**).

To determine the signaling target of p17 in the inhibition of the cGAS-STING pathway, p17 and the signaling molecules STING, TBK1, IKKϵ and IRF3-5D were co-transfected into 293T cells and downstream promoter activities examined. The results showed that p17 could inhibit the STING, TBK1 and IKKϵ but not IRF3-5D mediated IFNβ promoter activity (**Figure 6G**). Similarly, p17 inhibited the STING, TBK1 and IKK mediated NF-κB promoter activation (**Figure 6H**). These results together suggested that ASFV p17 can inhibit the cGAS-STING pathway through its interaction with STING and interference of the recruitment of TBK1 and IKKϵ, thus dampening not only STING signaling but also TBK1 and IKKϵ signaling.

### The Transmembrane (Amino Acids 39–59) of p17 Is Required for Its Interaction With STING and Inhibition of cGAS-STING Pathway

**Figure 7:**
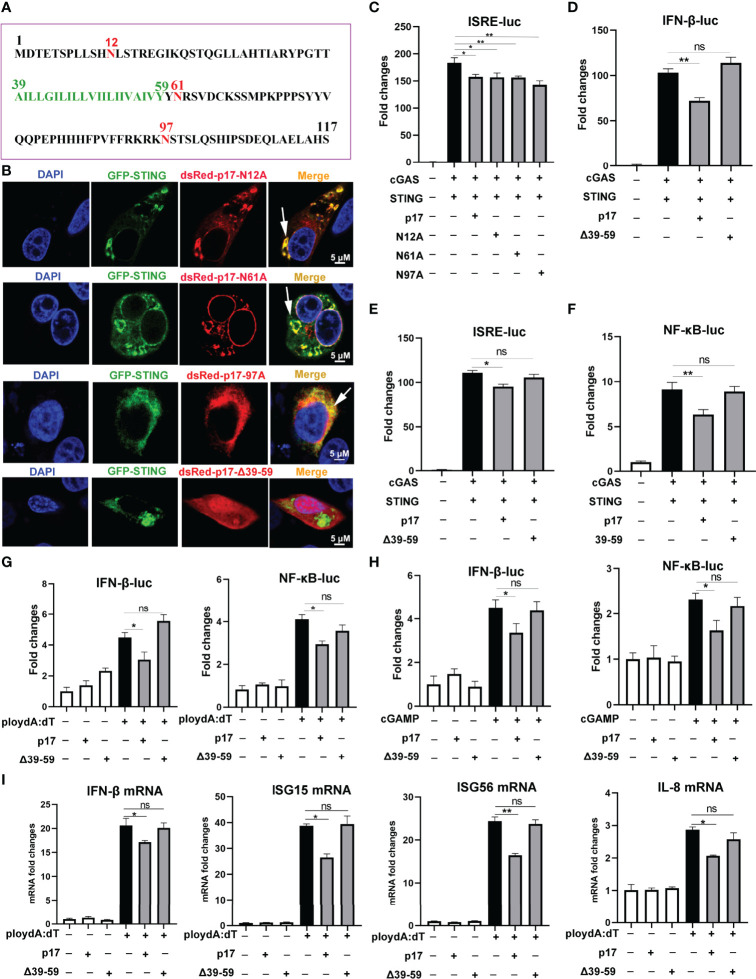
The effects of p17 mutants on cGAS-STING signaling pathway. **(A)** The protein amino acid sequence of p17, which consists of 117 amino acids and includes three glycosylation sites (red marked N12, N17 and N97) and one transmembrane domain (green marked A39-Y59). **(B)** The co-localizations between STING and p17 N12A, N17A, N97A, △39-59 in transfected 3D4/21 cells were examined by confocal microscopy. The arrows represent the co-localized punctate patterns. **(C)** The effects of p17 N12A, N17A and N97A on the ISRE promoter activation triggered by transfected porcine cGAS-STING in 293T cells were measured by Double-luciferase reporter assay. **(D–F)** The effects of p17 △39-59 on the promoter activations of IFN-β, ISRE and NF-κB triggered by cGAS-STING pathway in transfected 293T cells. **(G, H)** The effects of △39-59 on the activations of promoter IFN-β and NF-κB triggered by transfection of polydA:dT (1 μg/mL) and cGAMP (2 μg/mL) in 3D4/21 cells. **(I)** The effect of △39-59 on the polydA:dT activated mRNA expressions of IFN-β, ISG15, ISG56 and IL-8 were assayed by RT-qPCR in 3D4/21 cells. Values represent the mean ± S.D. *P < 0.05, **P < 0.01, ns not significant.

The protein sequence analysis of p17 from Uniprot website (https://www.uniprot.org) showed that there are three glycosylation sites including N12, N17 and N97, and one transmembrane domain (A39-Y59) in p17 (**Figure 7A**). To pinpoint the individual roles of each functional sites in the inhibitory activity, three point mutants N12A, N17A and N97A, and one deletion of amino acids 39-59 (△39-59) were made. The results from IFA showed that three mutants N12A, N17A and N97A co-localized with STING, but △39-59 had no co-localization with STING (**Figure 7B**, and **Supplementary Figure 3D**). The promoter assay in 293T cells showed that three mutants N12A, N17A and N97A could inhibit the activation of ISRE promoter (**Figure 7C**), whereas △39-59 had no effect on the activation of IFNβ, ISRE and NF-κB promoters induced by cGAS-STING pathway (**Figures 7D–F**). Similarly, the results from 3D4/21 cells have indicated that △39-59 had no effects on the activation of IFN-β and NF-κB promoters induced by both polydA:dT and 2’3’-cGAMP, respectively (**Figures 7G, H**). The effects of △39-59 on the polydA:dT activated cellular gene transcriptions were analyzed by RT-qPCR, and downstream mRNA expressions of IFN-β, ISG15, ISG56 and IL-8 were affected by p17 but not by mutant △39-59 (**Figure 7I**). These data suggested that the transmembrane of p17 is required for the co-localization with STING and the inhibition of cGAS-STING pathway.

**Figure 8:**
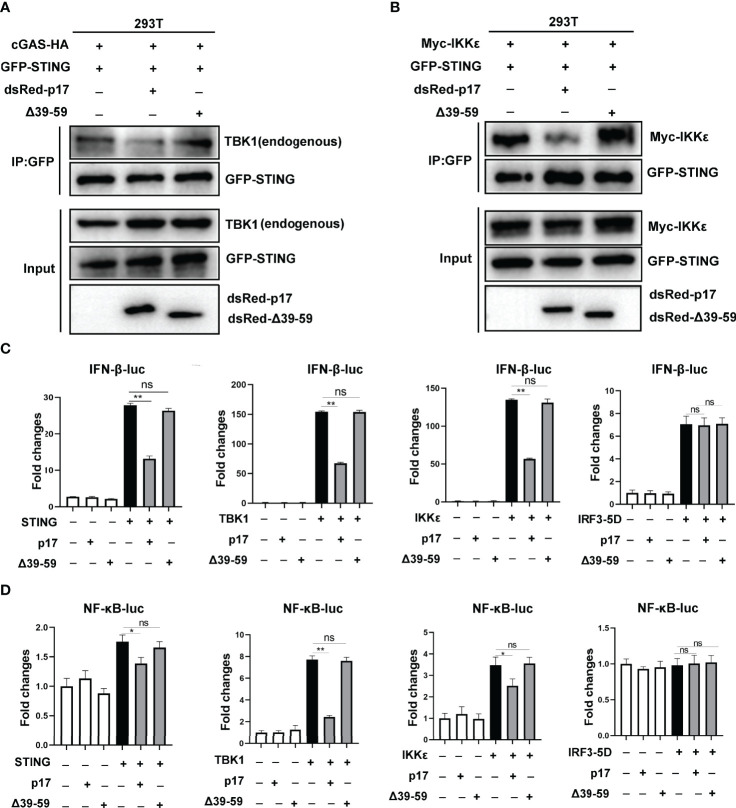
**(A, B)** The effect of p17 △39-59 on the binding between STING and TBK1/IKKϵ. **(A)** 293T cells in 6 well plate (6-8×10^5^ cells/well) were transfected with cGAS-HA (0.5 μg) and GFP-STING (0.5 μg) plus dsRed-p17 (0.5 μg) or △39-59 (0.5 μg) for 24h. **(B)** 293T cells were transfected with GFP-STING (0.5 μg) and Myc-IKKϵ (0.5 μg) plus dsRed-p17 (0.5 μg) or △39-59 (0.5 μg) for 24h. The effect of p17 △39-59 on the binding between STING and TBK1/IKKϵ was analyzed by Co-IP and Western blotting using the indicated antibodies. **(C, D)** The effects of p17 △39-59 on the activations of IFN-β **(C)** and NF-κB **(D)** promoter triggered by STING (20 ng), TBK1 (20 ng), IKKϵ (20 ng) and IRF3-5D (20 ng) were examined using dual-luciferase reporter assay. Values represent the mean ± S.D. *P < 0.05, **P < 0.01, ns not significant.

We further investigated the impact of mutant △39-59 on the interaction of STING and TBK1/IKKϵ and the signaling triggered by these molecules. The Co-IP results showed that, compared with p17, the △39-59 lost the ability to disturb the interaction of STING and TBK1 (**Figure 8A**), the interaction of STING and IKKϵ (**Figure 8B**). Consistently, relative to p17, mutant △39-59 also lost the ability to inhibit the signaling activities triggered by STING, TBK1 and IKKϵ in IFN-β promoter assay (**Figure 8C**) and in NF-κB promoter assay (**Figure 8D**).

**Figure 9:**
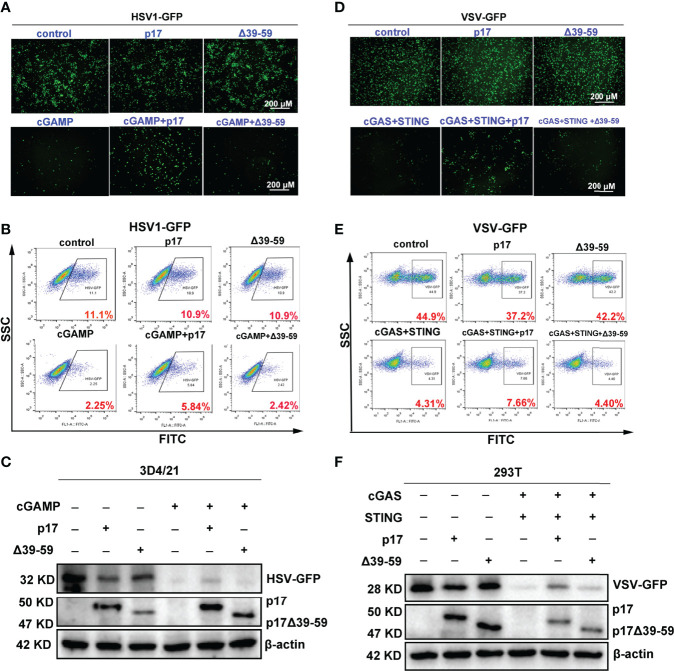
Effects of p17 △39-59 on the cGAS-STING signaling mediated anti-HSV1 and anti-VSV responses. **(A-C)** The p17 (0.5 μg) or △39-59 (0.5 μg) was transfected into 3D4/24 cells for 12 h, and the stimulated with transfection of 2’3’-cGAMP (2 μg/mL) for 12h. The cells were infected with HSV1-GFP at the MOI of 0.01 for 20 h. The HSV1 replicative GFP signals were visualized by fluorescence microscopy **(A)**, the HSV1-GFP infected cells were analyzed by flow cytometry **(B)** and the viral GFP protein expressions were detected by Western blotting **(C)**. **(D–F)** The p17 (0.5 μg) or △39-59 (0.5 μg) plus porcine cGAS (0.5 μg) and STING (0.5 μg) were co-transfected into 293T cells for 12 h. The cells were infected with VSV-GFP at the MOI of 0.001 for 8 h. The VSV replicative GFP signals were visualized by fluorescence microscopy **(D)**, the infected cells were analyzed by flow cytometry **(E)** and the viral GFP protein expressions were analyzed by Western blotting **(F)**.

Additionally, we found that △39-59 lost the ability to inhibit the 2’3’-cGAMP-STING signaling mediated anti-HSV1 activity in 3D4/21 cells, as evidenced by fluorescence microscopy (**Figure 9A**), flow cytometry (**Figure 9B**) and Western blotting (**Figure 9C**). Similarly, in 293T cells, △39-59 lost the ability to inhibit the porcine cGAS-STING signaling mediated anti-VSV activity, as evidenced by fluorescence microscopy (**Figure 9D**), flow cytometry (**Figure 9E**) and Western blotting (**Figure 9F**). Taken together, all these data suggested that the transmembrane of p17 is required for its STING interaction, disruption of the interaction between STING and TBK1/IKKϵ, and thus suppression of the triggered signaling activates and antiviral responses.

### Analyzing the Amino Acid Sequences Responsible for the Co-Localization Between ASFV p17 and STING

In order to minimize the functional sequence in the transmembrane (amino acids 39–59) of p17, more deletion mutants of p17 in pdsRed-Express-C1, including Δ39-48, Δ44-53, Δ49-59, Δ39-43, Δ44-48, Δ49-53 and Δ53-59 were made. The results from IFA showed that Δ39-48, Δ44-53, Δ49-59 and Δ53-59 had no co-localization with STING protein as Δ39-59. However, Δ39-43, Δ44-48, Δ49-53 were still co-localized with STING protein (**Supplementary Figures 2A, D**), indicating that within amino acids 39-59 of p17, most sequences longer than 5 amino acids are likely required for p17 co-localization with STING.

On the other hand, the roles of each functional domains of porcine STING in the interaction with ASFV p17 were also examined by IFA. The porcine STING protein includes four functional domains which are N-terminal domain mediating interaction with ZDHHC1 and ZDHHC11 (AAs 1-190), cyclic dinucleotide-binding domain (AAs 153-339), c-di-GMP binding domain (AAs 238-241) and C-terminal tail (AAs 339-378) (**Supplementary Figure 2B**). Four deletion pSTING mutants △1-190, △153-339, △238-241 and △339-378, and three individual pSTING fragments 1-190, 153-339 and 339-378 were obtained as pEGFP-C1 recombinant vectors, respectively. The con-focal microscopy results showed that △238-241 and △339-378 were co-localized with ASFV p17, however, △1-190, △153-339 and all three individual fragments lost the co-localization with ASFV p17. These data indicated the N-terminal domain (AAs 1-190) and middle domain (AAs 153-339) of STING are both required for the co-localization with ASFV p17 and STING (**Supplementary Figures 2C, E**).

### The Role of p17 in ASFV Caused IFN Evasion

**Figure 10:**
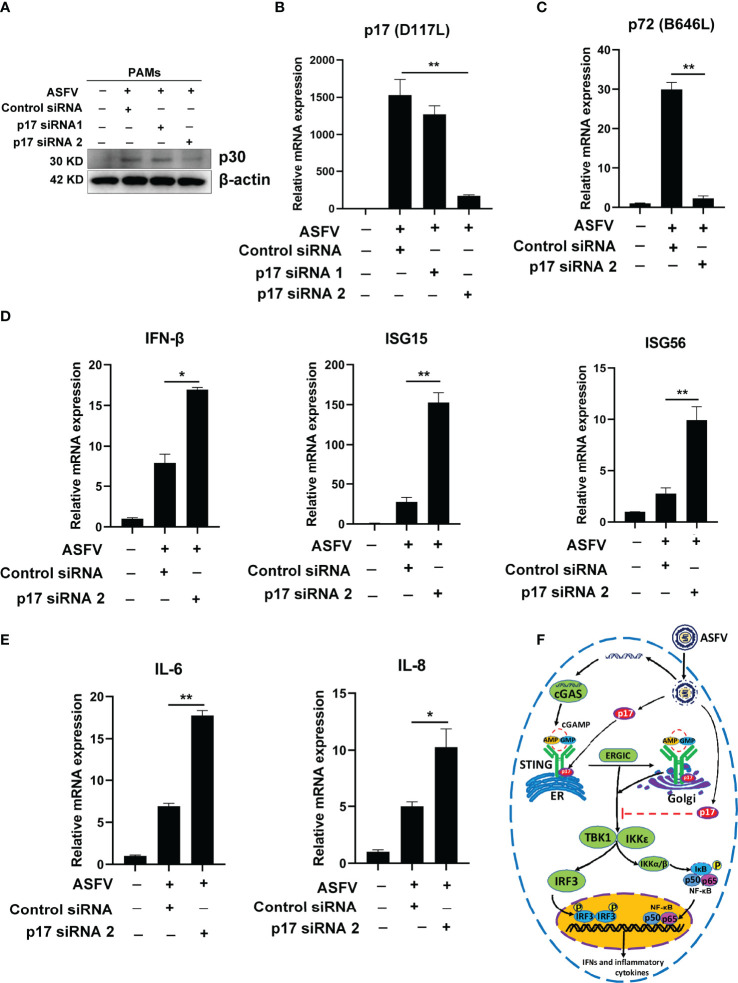
The role of p17 in ASFV caused IFN evasion. Primary PAMs (1×10^6^ cells/well) were transfected with various siRNAs (100 nM) for 24h and then infected with 0.01 MOI ASFV for another 72h. **(A)** The expression of ASFV p30 was detected by using Western blotting. **(B, C)** The transcriptions of viral p17 (D117L) and p72 (B646L) gene were analyzed by using RT-qPCR. **(D, E)** The effects of p17 on the host gene transcriptions of IFN-β, ISG15, ISG56, IL-6 and IL-8 induced by ASFV were analyzed by RT-qPCR. **(F)** The schematic presentation of the molecular mechanism of p17 inhibiting cGAS-STING pathway. Values represent the mean ± S.D. *P < 0.05, **P < 0.01.

Because p17 is essential for ASFV replication (16), it is not possible to test its immune evasion function using p17 gene deletion ASFV. Thus, in order to explore the role of p17 in the IFN induction during ASFV infection, p17 siRNA was used to knockdown the p17 expression in ASFV-infected primary PAMs. Two p17 siRNA were designed and the results from Western blotting and qPCR suggested the siRNA 2 is the effective one (**Figures 10A–C**). Compared to the control siRNA groups, the expressions of p17 gene and p30 protein were both decreased in p17 siRNA 2 treated cells (**Figures 10A, B**). The qPCR analysis further revealed that the transcription of viral p72 (B646L) gene was also decreased in p17 siRNA 2 treated cells (**Figure 10C**). Importantly, the qPCR results showed that relative to control siRNA groups, the ASFV induced gene transcriptions of IFN-β, ISG15, ISG56, IL-6 and IL-8 were all upregulated in p17 siRNA 2 groups (**Figures 10D, E**). These data suggested that knockdown the expression of p17 could reduce viral infection and promote the expression of IFN-related genes.

## Discussion

Most viral infections trigger a series of signaling cascades leading to the expression of type I IFNs, which exerts key roles in cellular antiviral responses (29, 30). The cGAS-STING DNA sensing pathway has been reported to play a pivotal role in inducing the production of IFNs and suppressing the replication of viruses, in particular DNA viruses (31). On the contrary, viruses have developed multiple strategies to resist host immune defenses, and counteract IFN signaling. ASFV encodes more than 150 polypeptides, which establish complex interactions with the host for the benefit of the virus in order to evade the host’s defenses (1). However, currently, there is still a lack of information regarding the roles of the viral proteins in host cells. In the current study, our results demonstrated that ASFV structural protein p17 exerts a negative regulatory effect on cGAS-STING signaling pathway and the STING signaling dependent anti-HSV1, anti-VSV and anti-ASFV functions.

Recently, an increasing number of studies have suggested that several proteins encoded by ASFV including A137R (32), D345L (33), DP96R (34), I215L (12), I226R (35), UBCv1 (15), S273R (25), MGF360-11L (36), MGF360-14L (37), MGF505-11R (38) and MGF-505-7R (39) have the ability to inhibit the cGAS-STING signaling pathway through different mechanisms. A137R, D345L, DP96R, I215L, S273R, MGF-505-7R and MGF360 could inhibit DNA sensing cGAS-STING pathway by targeting TBK1 or IKK kinase activity (12, 25, 32–34, 36, 39). I226R, MGF-505-7R and MGF360-14L negatively regulate cGAS-STING pathway by targeting IRF3 (35, 37, 39). MGF-505-7R and MGF505-11R could suppress the cGAS-STING pathway by interacting with STING (38, 39). UBCv1 manipulates the innate immune response *via* targeting the NF-κB and AP-1 pathways (15).These findings together with our current data have deepened our understanding of the immune escape mechanism of ASFV.

Accumulating evidences suggest that STING can be manipulated by viral products to counteract the cGAS-STING pathway and type I IFN production (40). STING, an ER-associated protein, is essential for inducing IFN in response to intracellular DNA or DNA pathogens including bacteria and DNA viruses (41, 42). In the presence of cytosolic DNA which binds with cGAS to produce 2’3’-cGAMP, STING dimer is subjected to self-oligomerization and sequential translocation from the ER to ERGIC, Golgi apparatus, and eventually to lysosome for degradation (43, 44). Upon translocation from ERGIC, STING recruits TBK1 to induce IFNs and other cytokines (45). The results from the current study suggested that ASFV p17 was located in ER and Golgi apparatus, and interacted with STING. ASFV p17 targets STING, disturbs the interaction of STING and TBK1, and thus inhibits IFN response. Furthermore, we found that ASFV p17 also disturbs the interaction of STING and IKKϵ. One recent study reported that IKKϵ and TBK1 act redundantly to activate STING mediated downstream NF-κB signaling (46). How significant the IKKϵ is in STING mediated IFN response and how significant the suppression of IKKϵ-STING interaction by p17 is in the ASFV immune evasion are not known and would need to be addressed in the future.

ASFV p17 protein is encoded by the D117L gene and consists of 117 amino acids. There are three glycosylation sites including N12, N61 and N97, and one transmembrane domain (AAs 39-59) (19). Our co-localization results indicated that the most transmembrane of p17 except amino acids 39-43 may be important for interaction with STING, whereas in STING, the N-terminal portion and middle cyclic dinucleotide-binding domain might be required for interaction with ASFV p17, and the C-terminal CTT domain of STING is not involved in the interaction. The CTT domain is the key portion for STING to recruit and activate TBK1 and IRF3, thus the disturbing the interaction of STING and TBK1 by p17 is likely through an indirect way. Finally, the ASFV infection was analyzed at 72 hpi, when the virus replication was significant while the cell viability was not obviously decreased. The results from siRNA experiments disclosed that knockdown the expression of p17 could reduce viral infection and promote the expression of IFN-related genes, confirming the role of p17 as an IFN immune evasion protein of ASFV.

In summary, this study revealed that ASFV p17 is able to inhibit the cGAS-STING pathway through interacting with STING and interfering STING to recruit TBK1 and IKKϵ (**Figure 10F**). The transmembrane domain of p17 is required for its interacting with STING and inhibiting cGAS-STING pathway. Findings in this study will expand our knowledge on the molecular mechanisms by which ASFV counteracts the antiviral innate immunity and provide deep insights into ASF pathogenesis.

## Data Availability Statement

The original contributions presented in the study are included in the article/**Supplementary Material**. Further inquiries can be directed to the corresponding author.

## Author Contributions

JZZ and WZ conceived and designed the experiments; WZ, NX, JJZ, QC, SJ, JL, HW performed the experiments; WZ, NC, QZ, FM, and JZZ analyzed the data; WZ and JZZ wrote the paper. All authors contributed to the article and approved the submitted version.

## Funding

The work was partly supported by the National Key Research and Development Program of China (2021YFD1800100), Jiangsu provincial key R & D plan (BE2020398), Jiangsu agricultural science and technology independent innovation fund project (CX(21)2035), the National Natural Science Foundation of China (32172867; 31872450), and A Project Funded by the Priority Academic Program Development of Jiangsu Higher Education Institutions (PAPD).

## Conflict of Interest

The authors declare that the research was conducted in the absence of any commercial or financial relationships that could be construed as a potential conflict of interest.

## Publisher’s Note

All claims expressed in this article are solely those of the authors and do not necessarily represent those of their affiliated organizations, or those of the publisher, the editors and the reviewers. Any product that may be evaluated in this article, or claim that may be made by its manufacturer, is not guaranteed or endorsed by the publisher.

## Supplementary Material

Supplementary Figure 1 The subcellular localization of p17 in cells. 3D4/21 cells (1.5×10^5^ cells/well) grown on glass coverslip in 24-well plate were transfected with p17 plasmids (0.5μg) together with RFP-KDEL (an ER retention protein) (0.5μg) **(A)**, with Golgi-RFP (sialyl transferase) (0.5μg) **(B)**, with Mito-EGFP (mitochondrial targeting sequence of cytochrome c oxidase subunit VII fused to EGFP) (0.5μg) **(C)**, with GFP-LAMP1 (lysosomal-associated membrane protein1) (0.5μg) **(D)**, respectively, as indicated. The FLAG-p17 was stained with rabbit anti-FLAG mAb and second antibody. The subcellular localization of p17 protein in cells was visualized by confocal fluorescence microscopy. **(E)** The co-localizations in multiple vision fields were analyzed using Image J, and the Pearson correlation coefficient values were graphed.

Supplementary Figure 2 Analyzing the amino acid sequences responsible for the co-localization between p17 and STING. **(A)** 3D4/21 cells on coverslips in 12-well plate (1.5×10^5^ cells/well) were co-transfected with mCherry-STING (0.5 μg) plus p17-GFP △39-59, △39-48, △44-53, △49-59, △39-43, △44-48, △49-53 or △53-59 (0.5 μg each) for 24 h. Cells were fixed, and then counter stained by DAPI. **(B)** Based on the protein function domains of porcine STING, a series of STING deletion mutants were constructed as indicated. **(C)** 3D4/21 cells on coverslips in 12-well plate (1.5×10^5^ cells/well) were co-transfected with dsRed-p17 (0.5 μg) plus GFP-pSTING △1-190, △153-339, △238-241, △339-378, 1-190, 153-339 or 339-378 (0.5 μg each) for 24 h, cells were fixed, and then counter stained by DAPI. Green fluorescence protein GFP and red fluorescence protein dsRed expression in 3D4/21 cells were directly visualized and cellular co-localizations detected by confocal microscopy. The arrows indicate the co-localization areas. **(D, E)** The co-localizations in multiple vision fields from A **(D)** and from C **(E)** were analyzed using Image J, and the Pearson correlation coefficient values were graphed.

Supplementary Figure 3 The co-localization quantifications for Fig 4 **(A)**, Fig 5G-H **(B)**, Fig 6E-F **(C)** and Fig 7B **(D)**, respectively. Multiple vision fields were analyzed using Image J, and the Pearson correlation coefficient values from 10 positive cells were graphed. The value of Pearson correlation coefficient reflects the level of co-localization, with 1.0 representing 100% co-localization.
