# Supplementary figures and images for: African Swine Fever Virus Structural Protein p17 Inhibits cGAS-STING Signaling Pathway Through Interacting With STING
Source: Front Immunol. 2022 Jul 1;13:941579. doi: 10.3389/fimmu.2022.941579 (PMC9283692; doi:10.3389/fimmu.2022.941579)

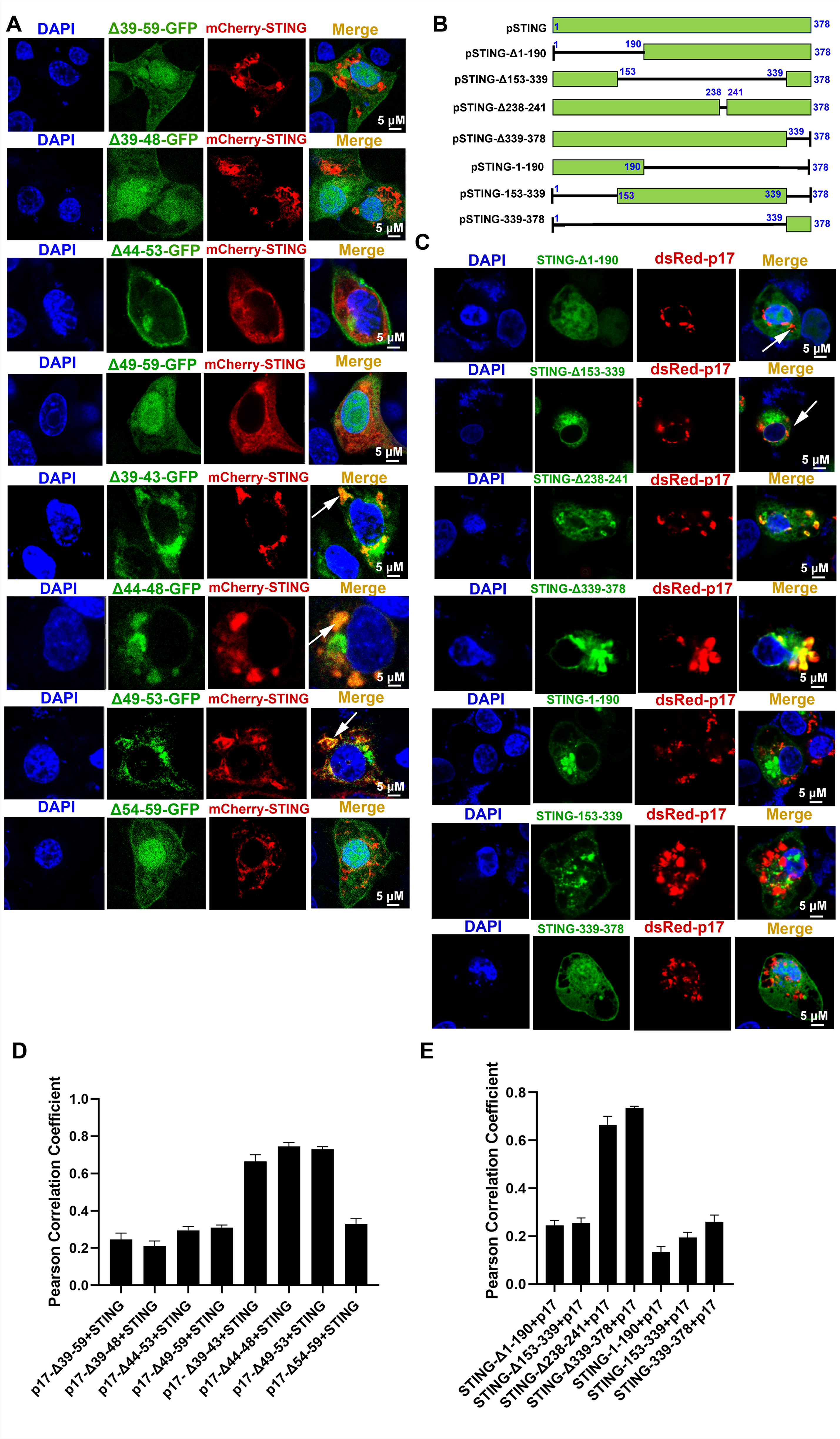

Supplement: Supplementary Figure 2 — Analyzing the amino acid sequences responsible for the co-localization between p17 and STING. (A) 3D4/21 cells on coverslips in 12-well plate (1.5×10^5^ cells/well) were co-transfected with mCherry-STING (0.5 μg) plus p17-GFP △39-59, △39-48, △44-53, △49-59, △39-43, △44-48, △49-53 or △53-59 (0.5 μg each) for 24 h. Cells were fixed, and then counter stained by DAPI. (B) Based on the protein function domains of porcine STING, a series of STING deletion mutants were constructed as indicated. (C) 3D4/21 cells on coverslips in 12-well plate (1.5×10^5^ cells/well) were co-transfected with dsRed-p17 (0.5 μg) plus GFP-pSTING △1-190, △153-339, △238-241, △339-378, 1-190, 153-339 or 339-378 (0.5 μg each) for 24 h, cells were fixed, and then counter stained by DAPI. Green fluorescence protein GFP and red fluorescence protein dsRed expression in 3D4/21 cells were directly visualized and cellular co-localizations detected by confocal microscopy. The arrows indicate the co-localization areas. (D, E) The co-localizations in multiple vision fields from A (D) and from C (E) were analyzed using Image J, and the Pearson correlation coefficient values were graphed. [file Image_2.tif]

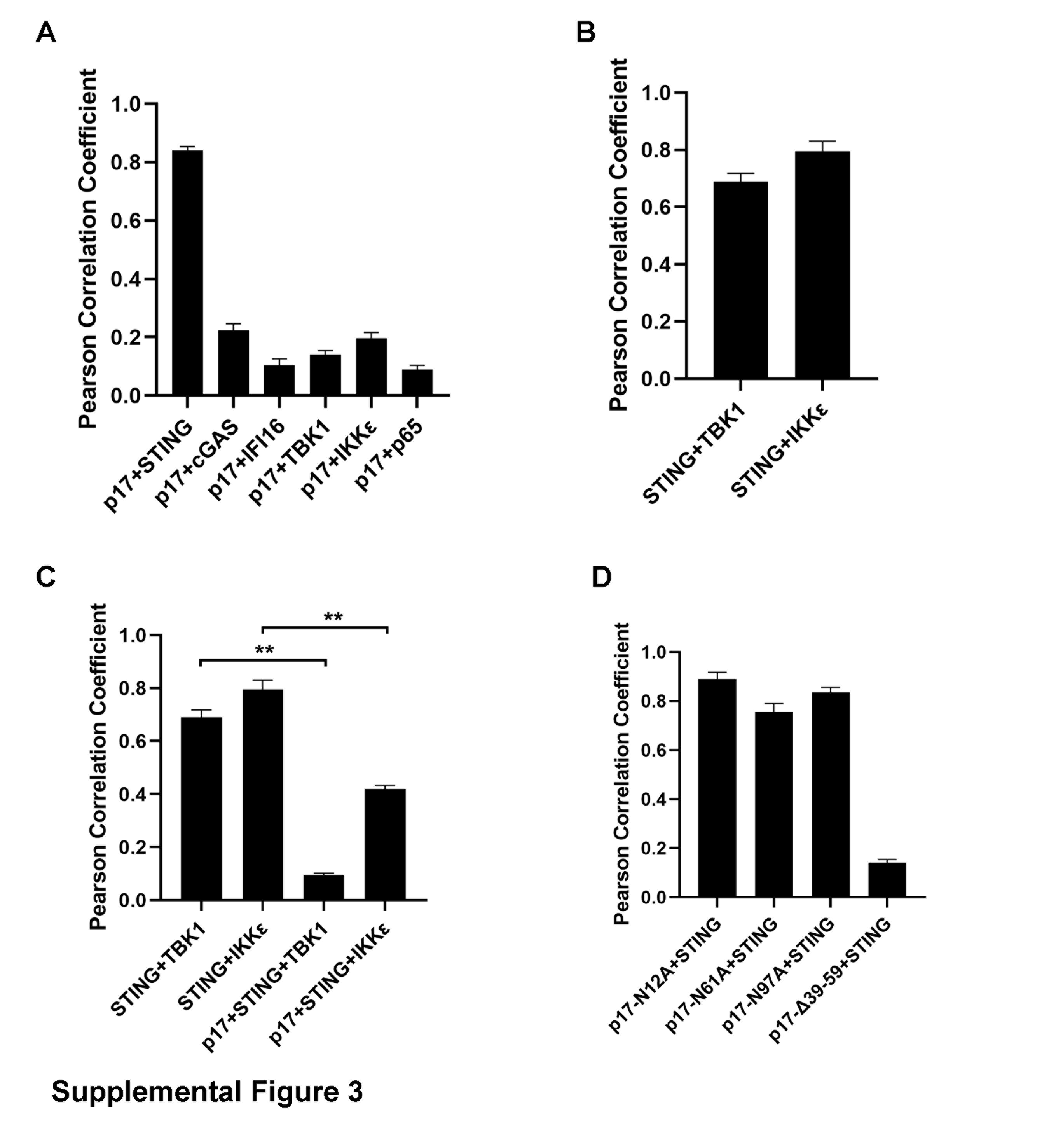

Supplement: Supplementary Figure 3 — The co-localization quantifications for Fig 4 (A), Fig 5G-H (B), Fig 6E-F (C) and Fig 7B (D), respectively. Multiple vision fields were analyzed using Image J, and the Pearson correlation coefficient values from 10 positive cells were graphed. The value of Pearson correlation coefficient reflects the level of co-localization, with 1.0 representing 100% co-localization. [file Image_3.tif]
