# Supplementary material for: African Swine Fever Virus Structural Protein p17 Inhibits cGAS-STING Signaling Pathway Through Interacting With STING
Source: Front Immunol. 2022 Jul 1;13:941579. doi: 10.3389/fimmu.2022.941579 (PMC9283692; doi:10.3389/fimmu.2022.941579)

Figure 1. F

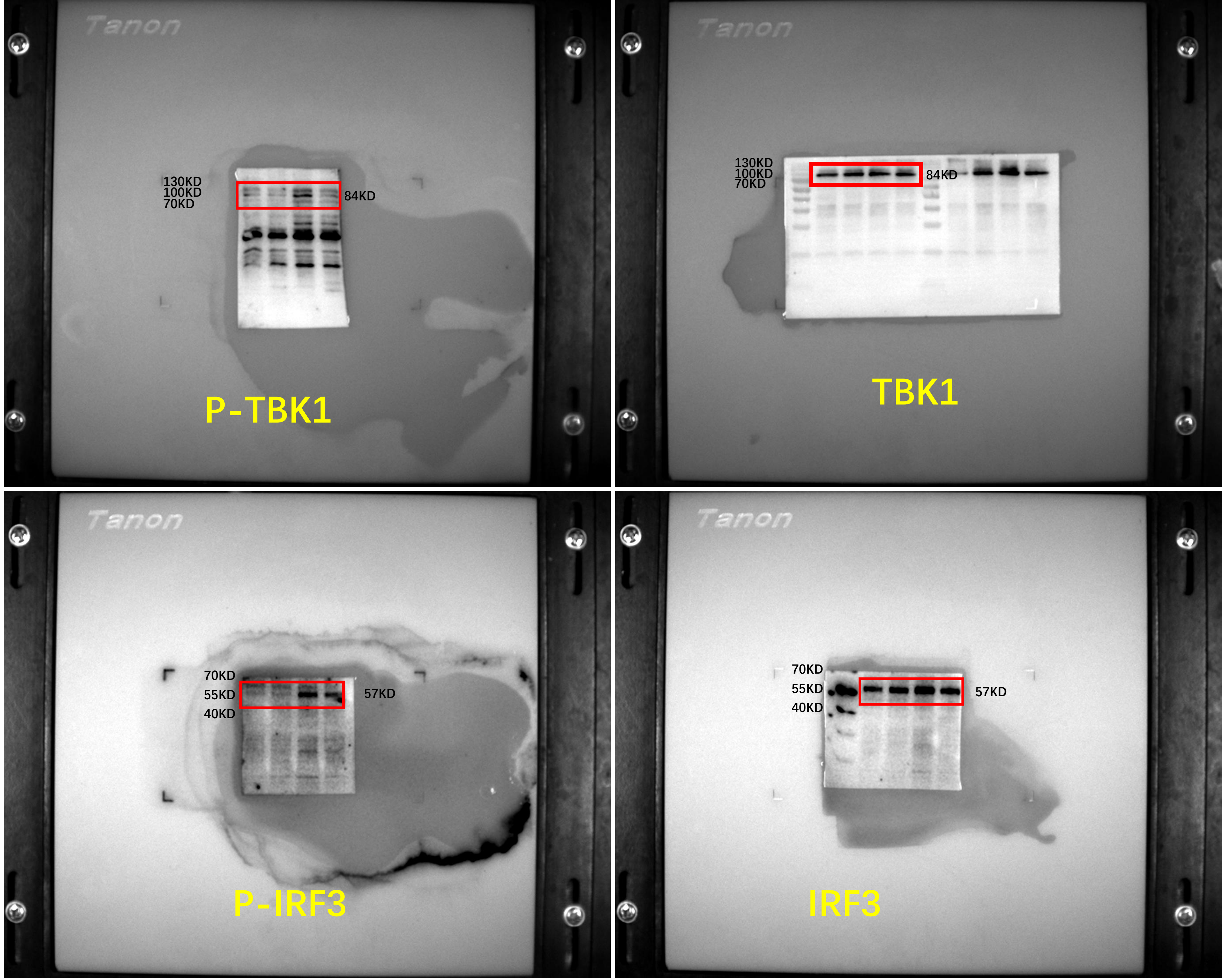

Tanon
130KD
100KD
70KD
84KD
P-TBK1
Tanon
130KD
100KD
70KD
84KD
TBK1
Tanon
70KD
55KD
40KD
57KD
P-IRF3
Tanon
70KD
55KD
40KD
57KD
IRF3

Figure 1. F

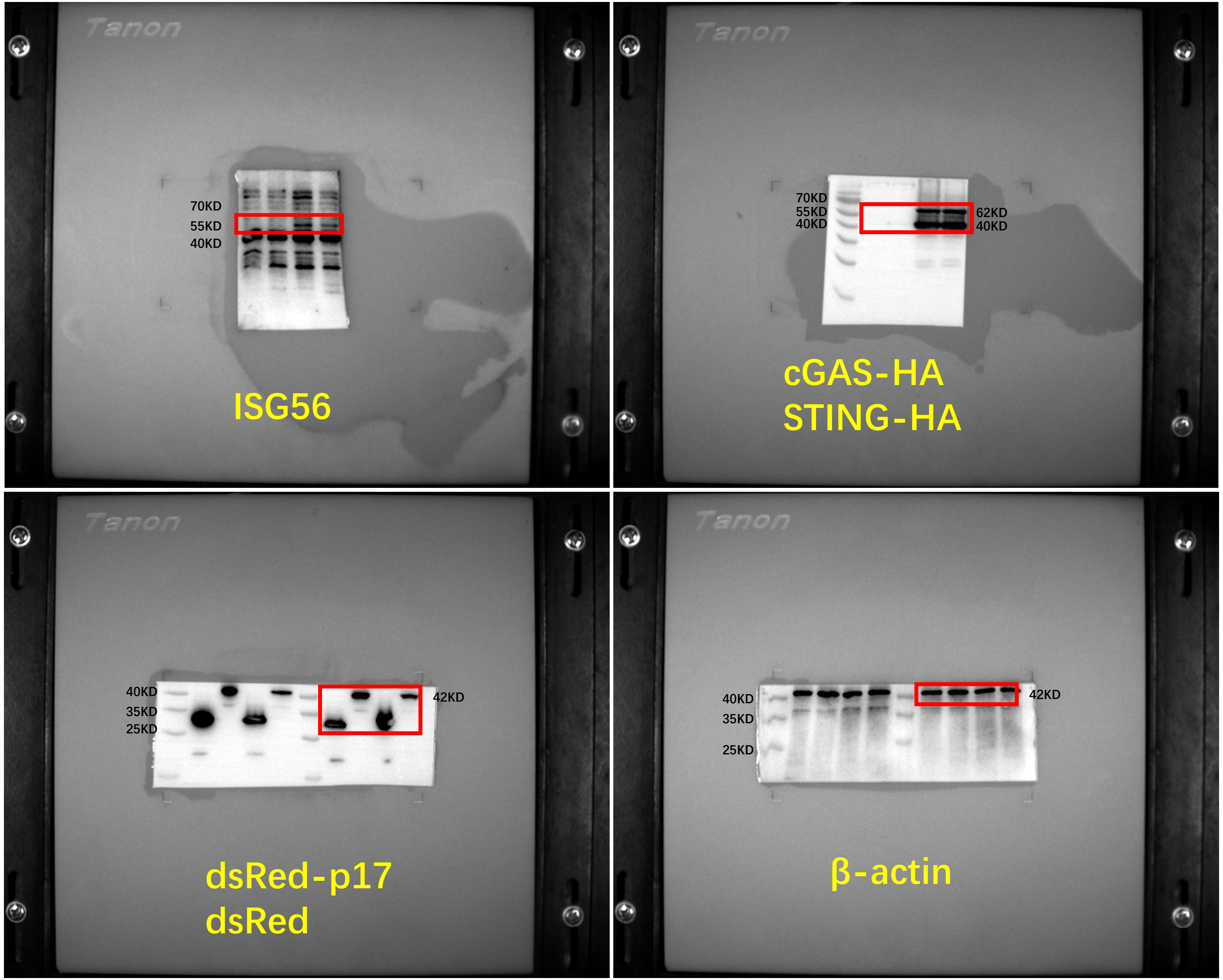

70KD
55KD
40KD
ISG56
70KD
55KD
40KD
62KD
40KD
cGAS-HA
STING-HA
40KD
35KD
25KD
42KD
dsRed-p17
dsRed
40KD
35KD
25KD
42KD
β-actin

Figure 1. G

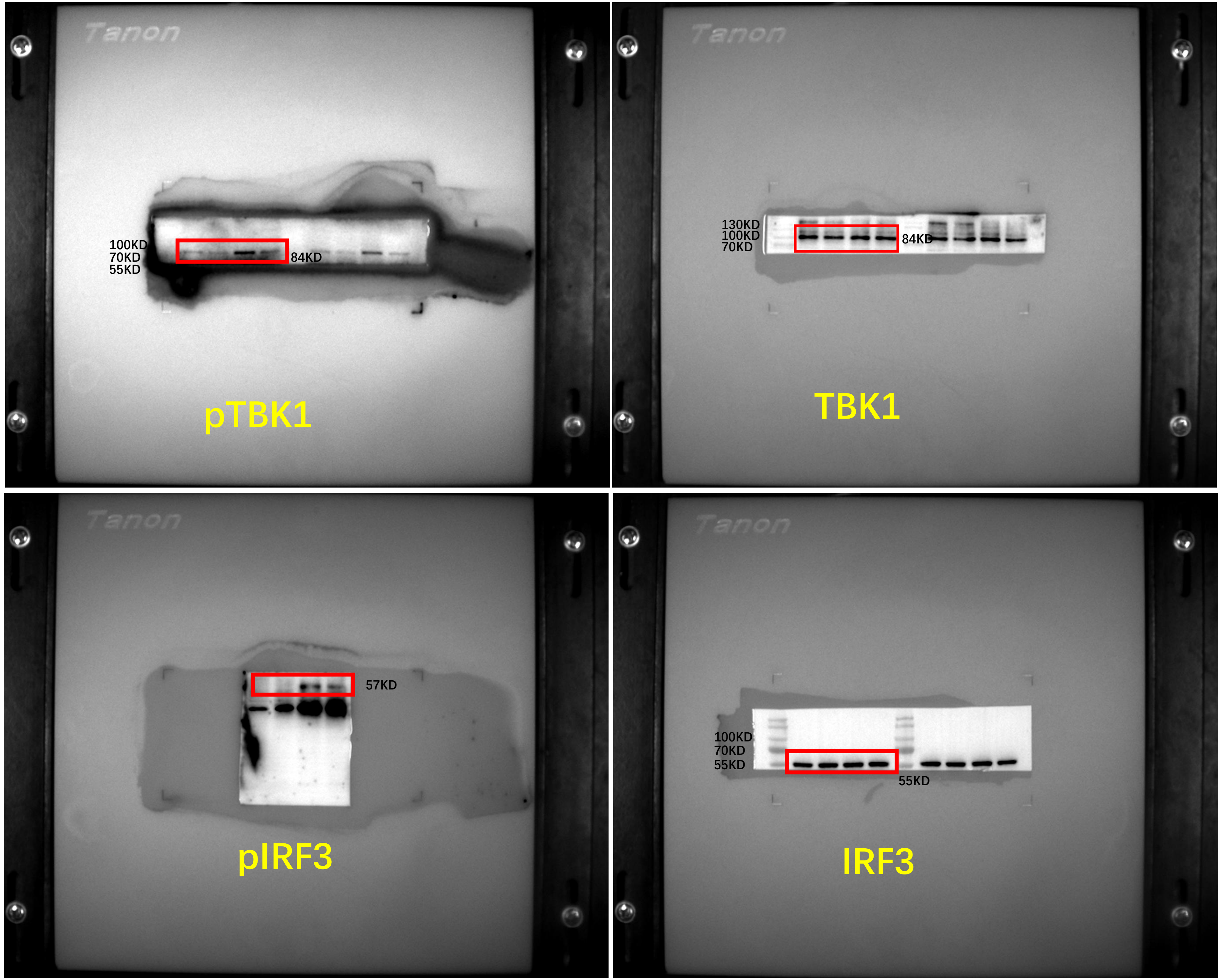
100KD
70KD
55KD
84KD
pTBK1
130KD
100KD
70KD
84KD
TBK1
57KD
pIRF3
100KD
70KD
55KD
55KD
IRF3

Figure 1. G

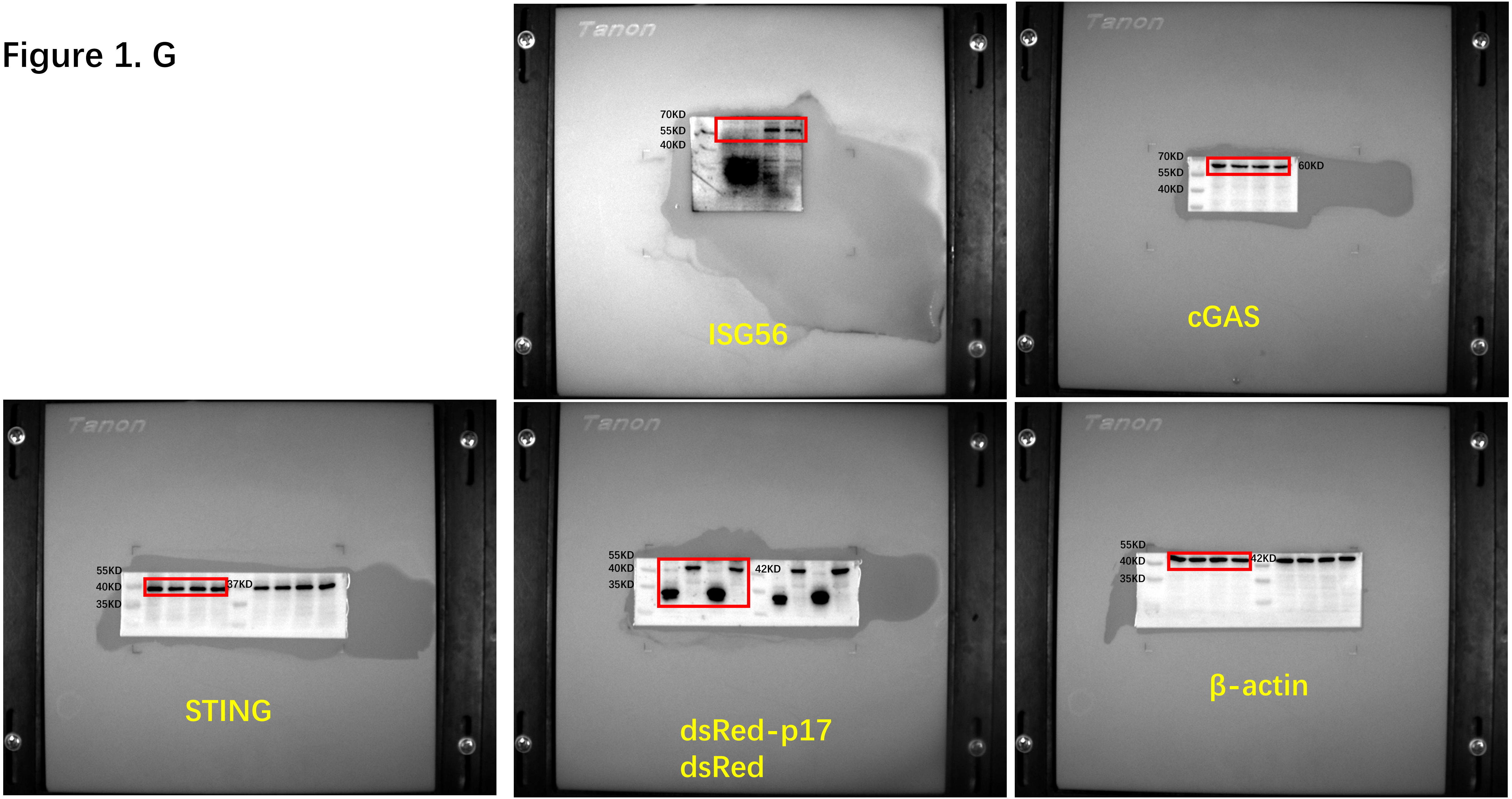

70KD
55KD
40KD
ISG56
70KD
55KD
40KD
60KD
cGAS
55KD
40KD
35KD
37KD
STING
55KD
40KD
35KD
42KD
dsRed-p17
dsRed
55KD
40KD
35KD
42KD
β-actin

Figure 1. H

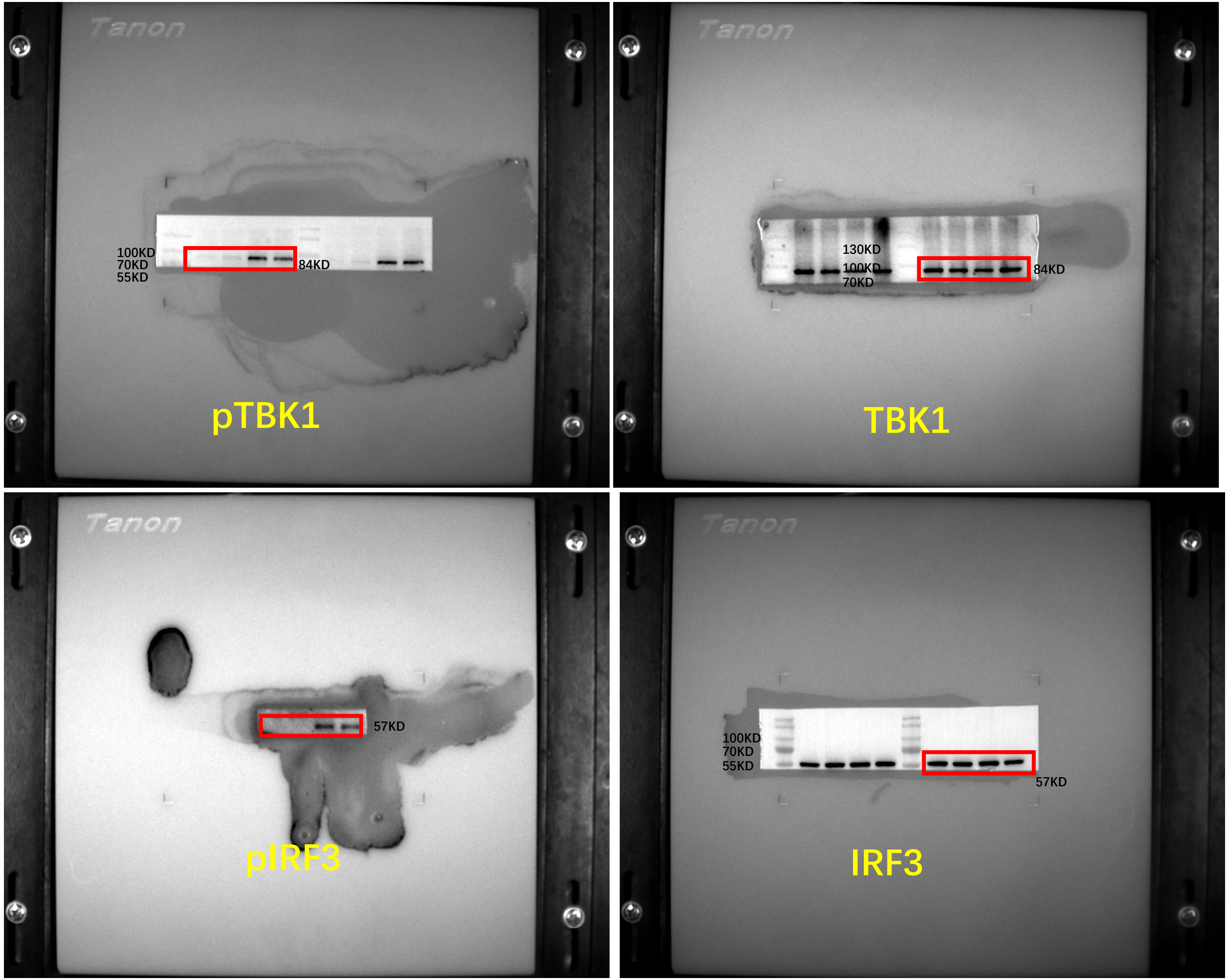

100KD
70KD
55KD
84KD
pTBK1
130KD
100KD
70KD
84KD
TBK1
57KD
pIRF3
100KD
70KD
55KD
57KD
IRF3

Figure 1. H

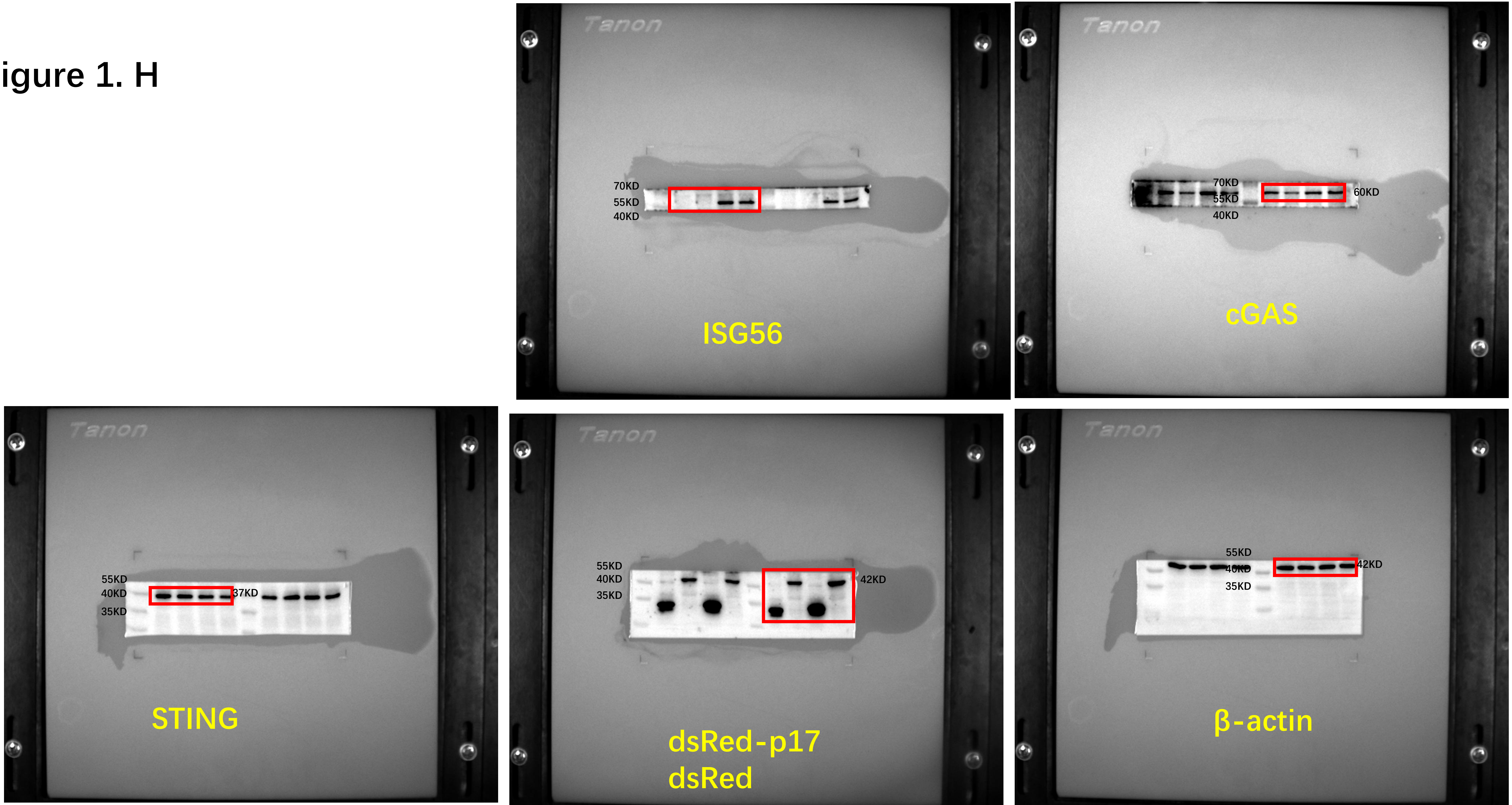
70KD
55KD
40KD
ISG56
70KD
55KD
40KD
60KD
cGAS
55KD
40KD
35KD
37KD
STING
55KD
40KD
35KD
42KD
dsRed-p17
dsRed
55KD
40KD
35KD
42KD
β-actin

Figure 2. F

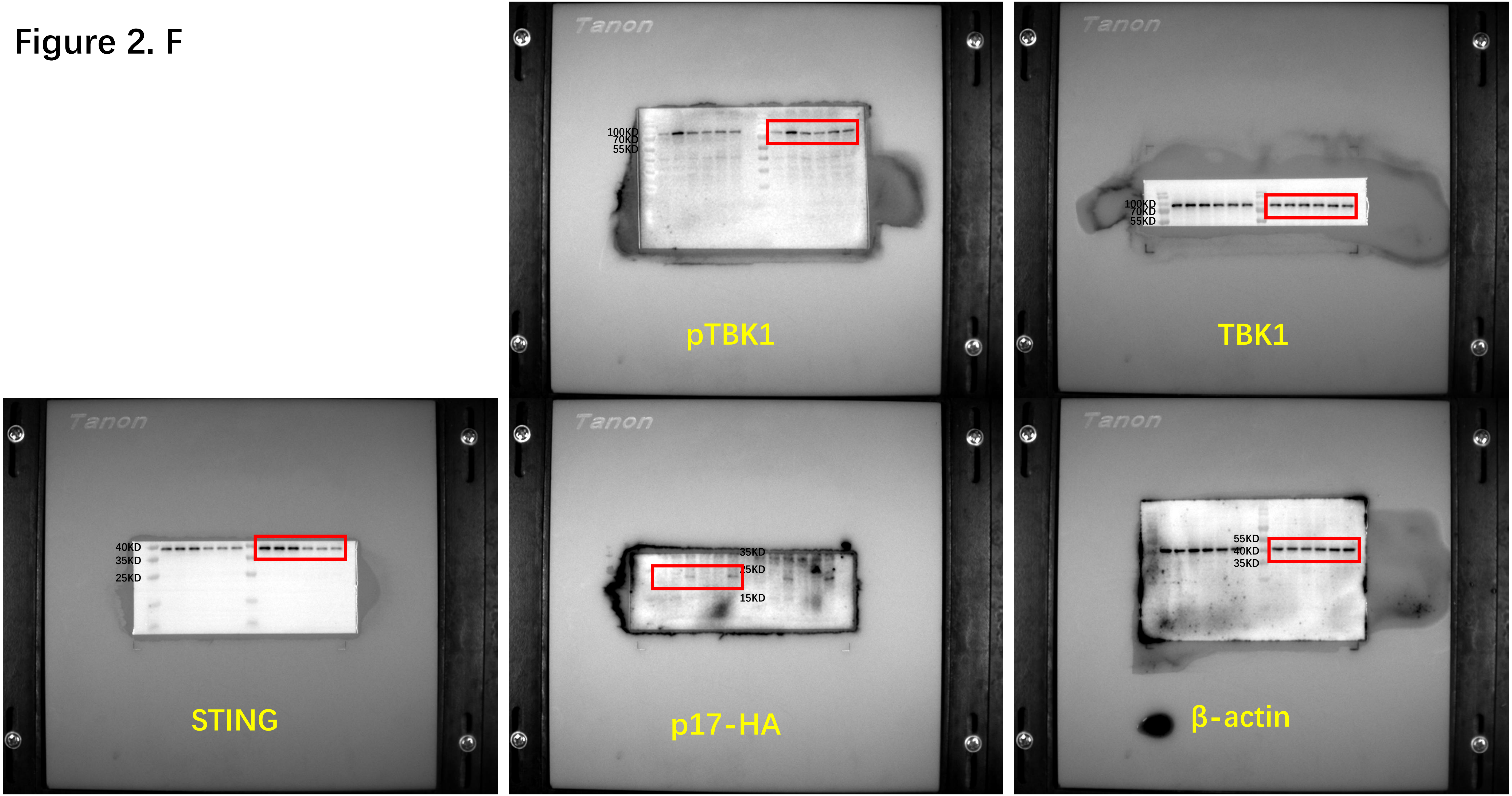
pTBK1
100KD
70KD
55KD
TBK1
100KD
70KD
55KD
STING
40KD
35KD
25KD
p17-HA
35KD
25KD
15KD
β-actin
55KD
40KD
35KD

Figure 3. C

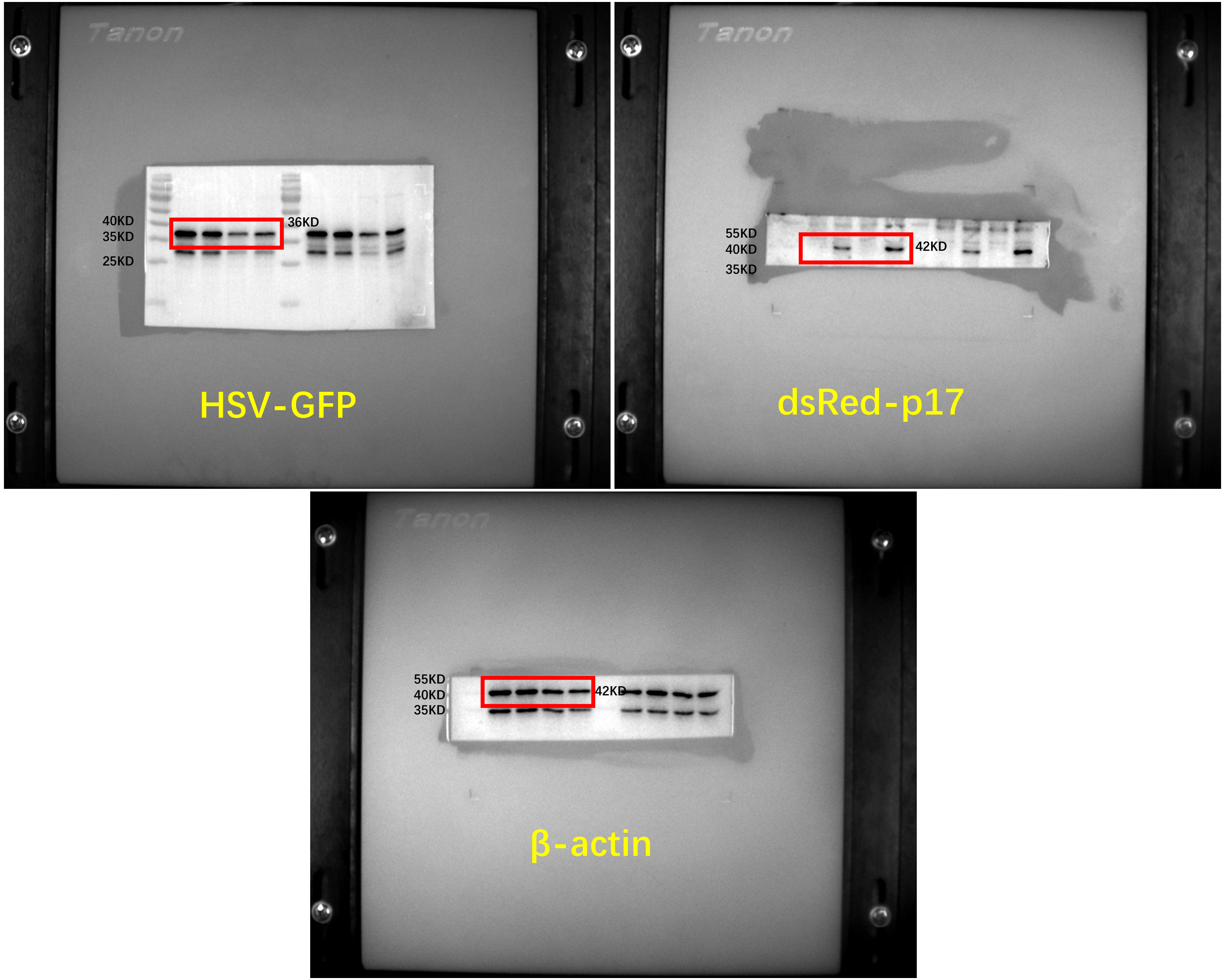
Tanon
40KD
35KD
25KD
36KD
HSV-GFP
Tanon
55KD
40KD
35KD
42KD
dsRed-p17
Tanon
55KD
40KD
35KD
42KD
β-actin

Figure 3. H

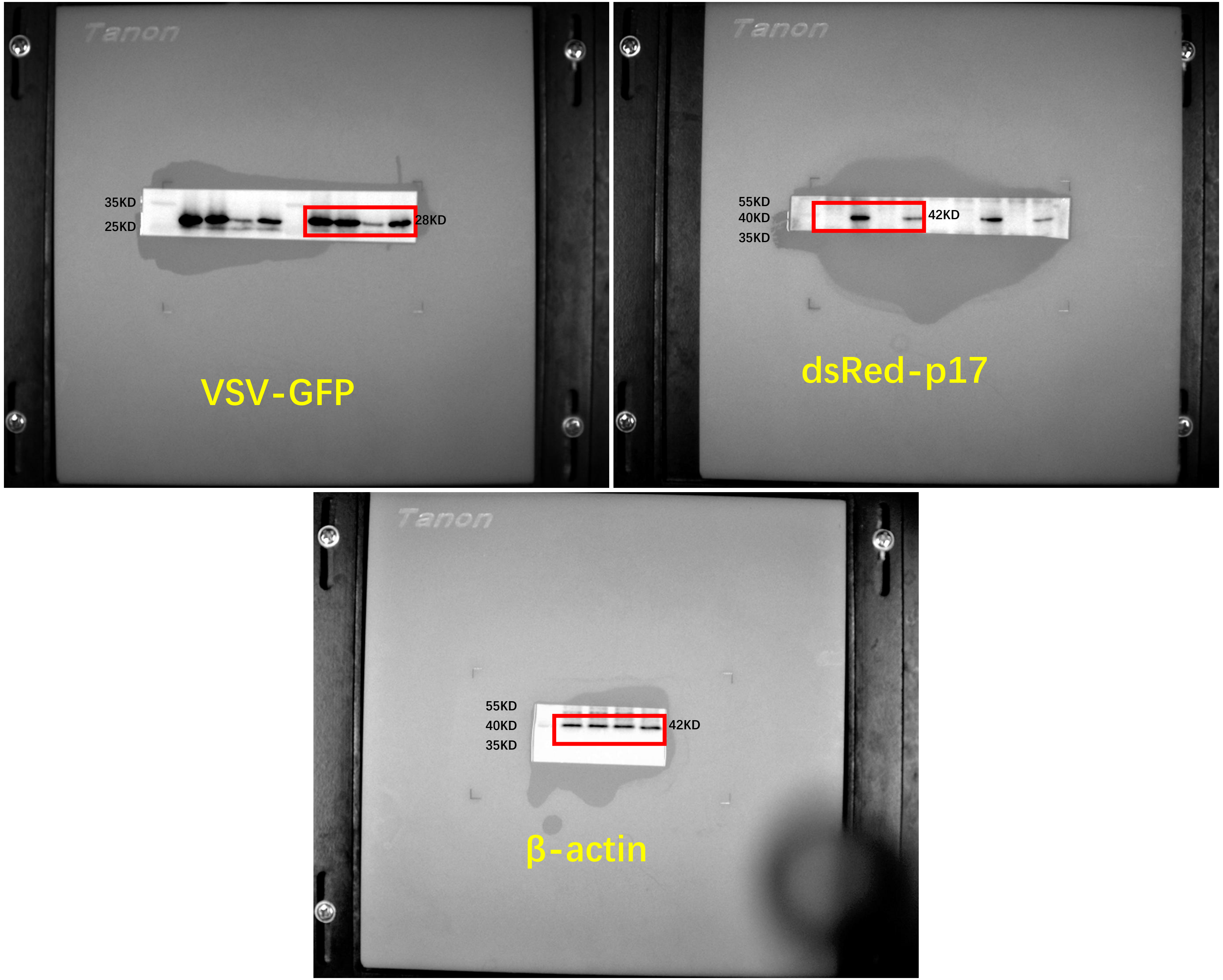
35KD
25KD
28KD
VSV-GFP
55KD
40KD
35KD
42KD
dsRed-p17
55KD
40KD
35KD
42KD
β-actin

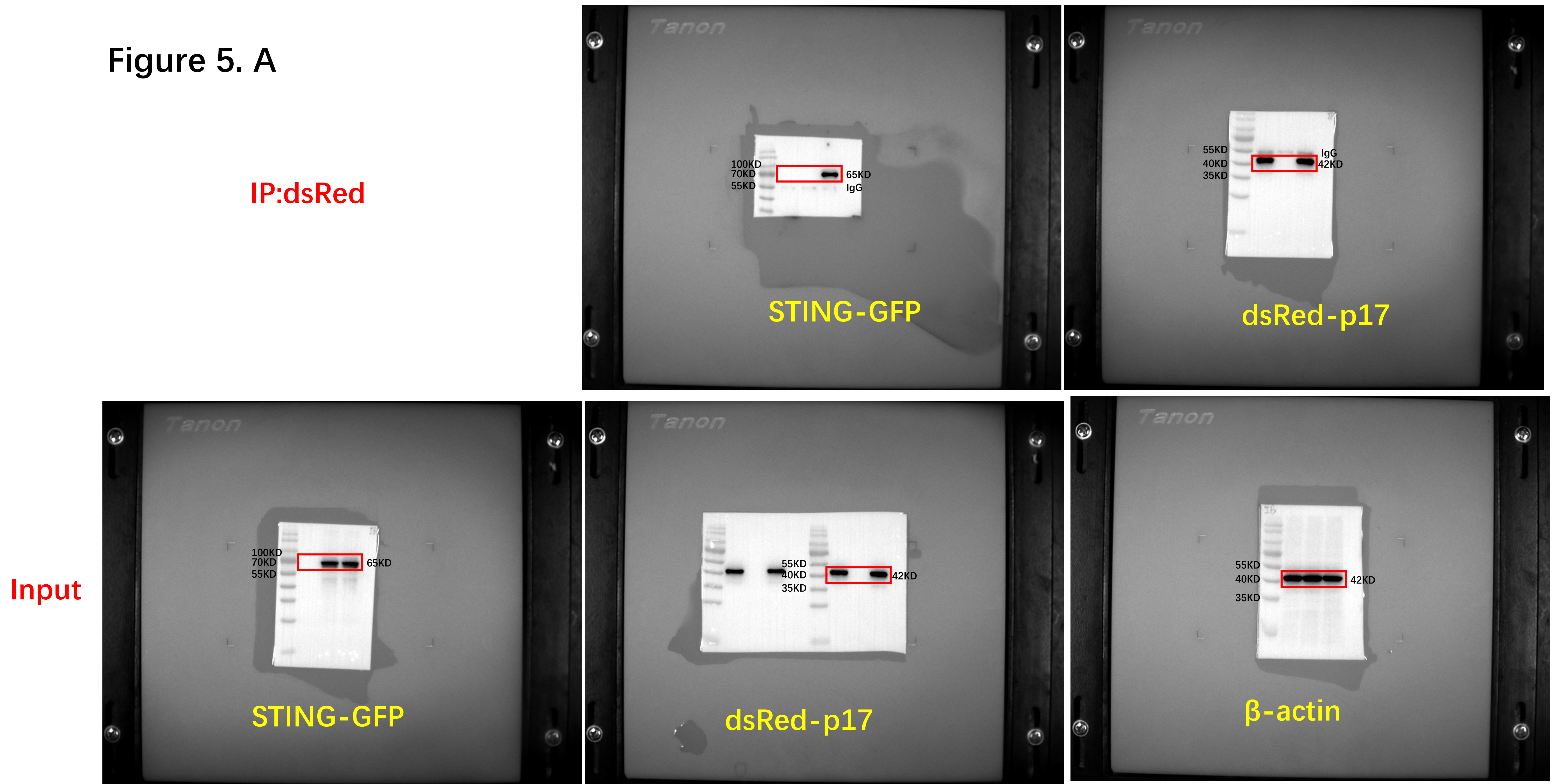
Figure 5. A
IP:dsRed
Input
100KD
70KD
55KD
65KD
IgG
STING-GFP
55KD
40KD
35KD
IgG
42KD
dsRed-p17
100KD
70KD
55KD
65KD
STING-GFP
55KD
40KD
35KD
42KD
dsRed-p17
55KD
40KD
35KD
42KD
β-actin

Figure 5. B

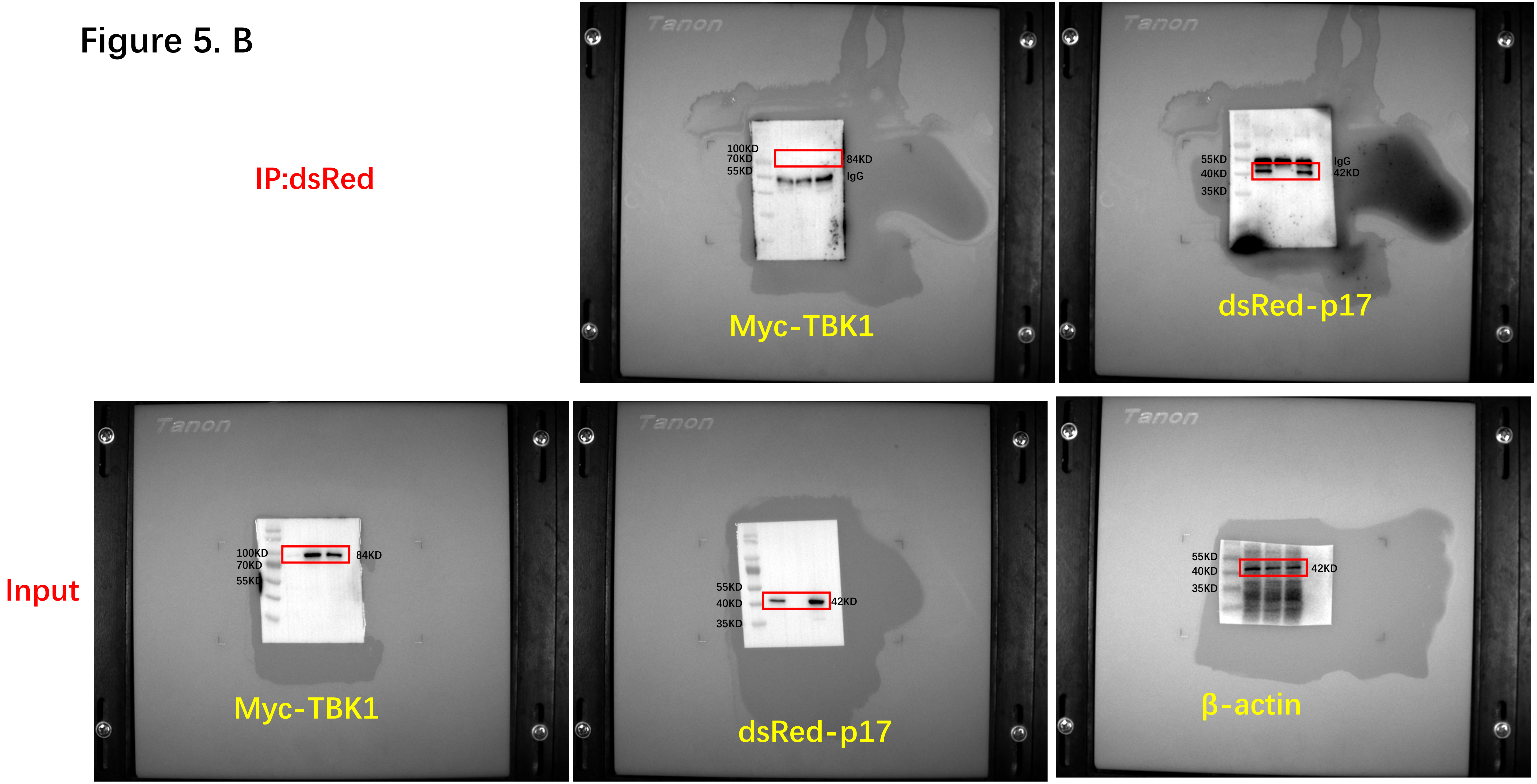

IP:dsRed
100KD
70KD
55KD
84KD
IgG
Myc-TBK1
55KD
40KD
35KD
IgG
42KD
dsRed-p17
Input
100KD
70KD
55KD
84KD
Myc-TBK1
55KD
40KD
35KD
42KD
dsRed-p17
55KD
40KD
35KD
42KD
β-actin

Figure 5. C

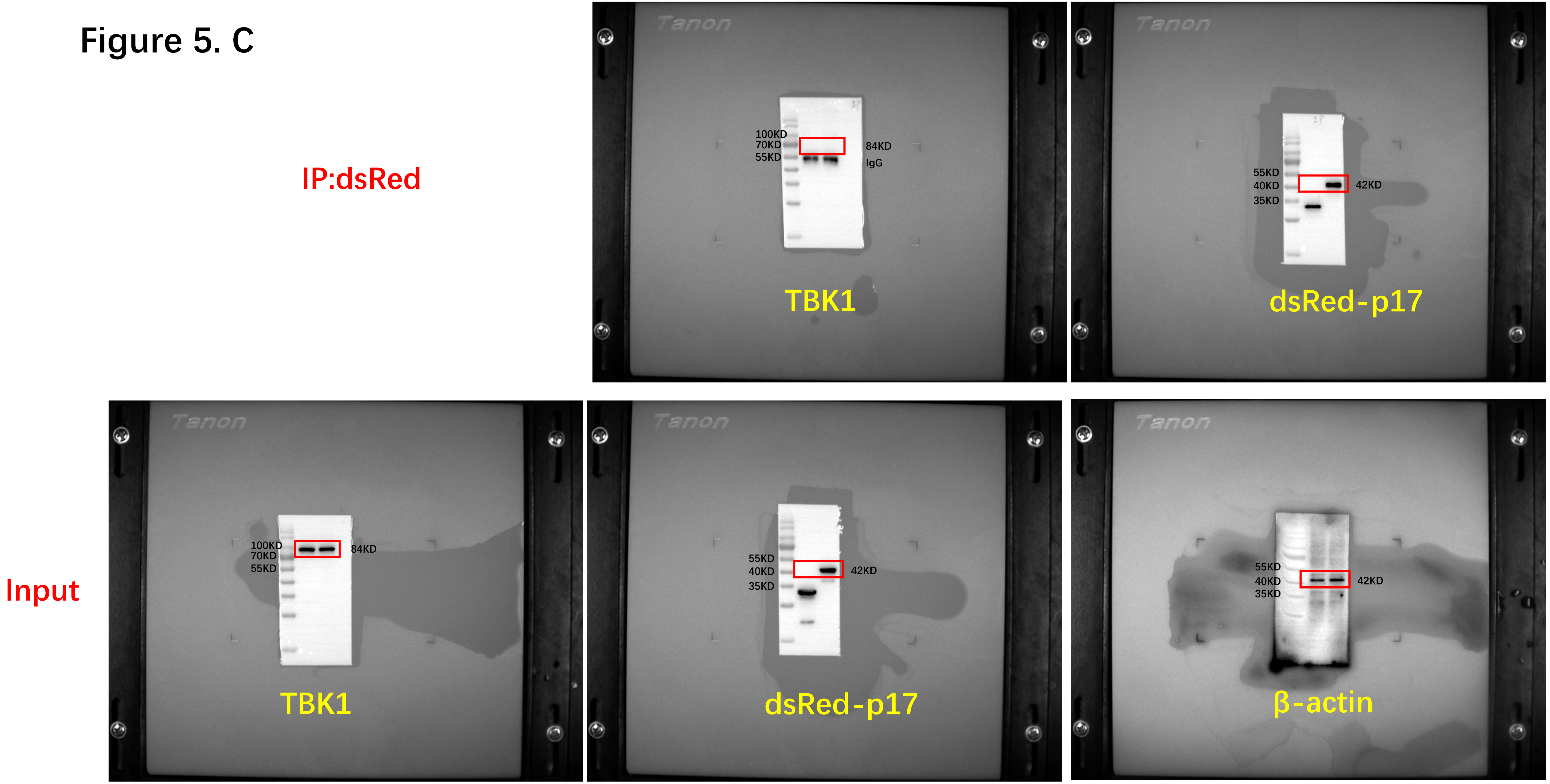
IP:dsRed
Input
100KD
70KD
55KD
84KD
IgG
TBK1
55KD
40KD
35KD
42KD
dsRed-p17
100KD
70KD
55KD
84KD
TBK1
55KD
40KD
35KD
42KD
dsRed-p17
55KD
40KD
35KD
42KD
β-actin

Figure 5. D

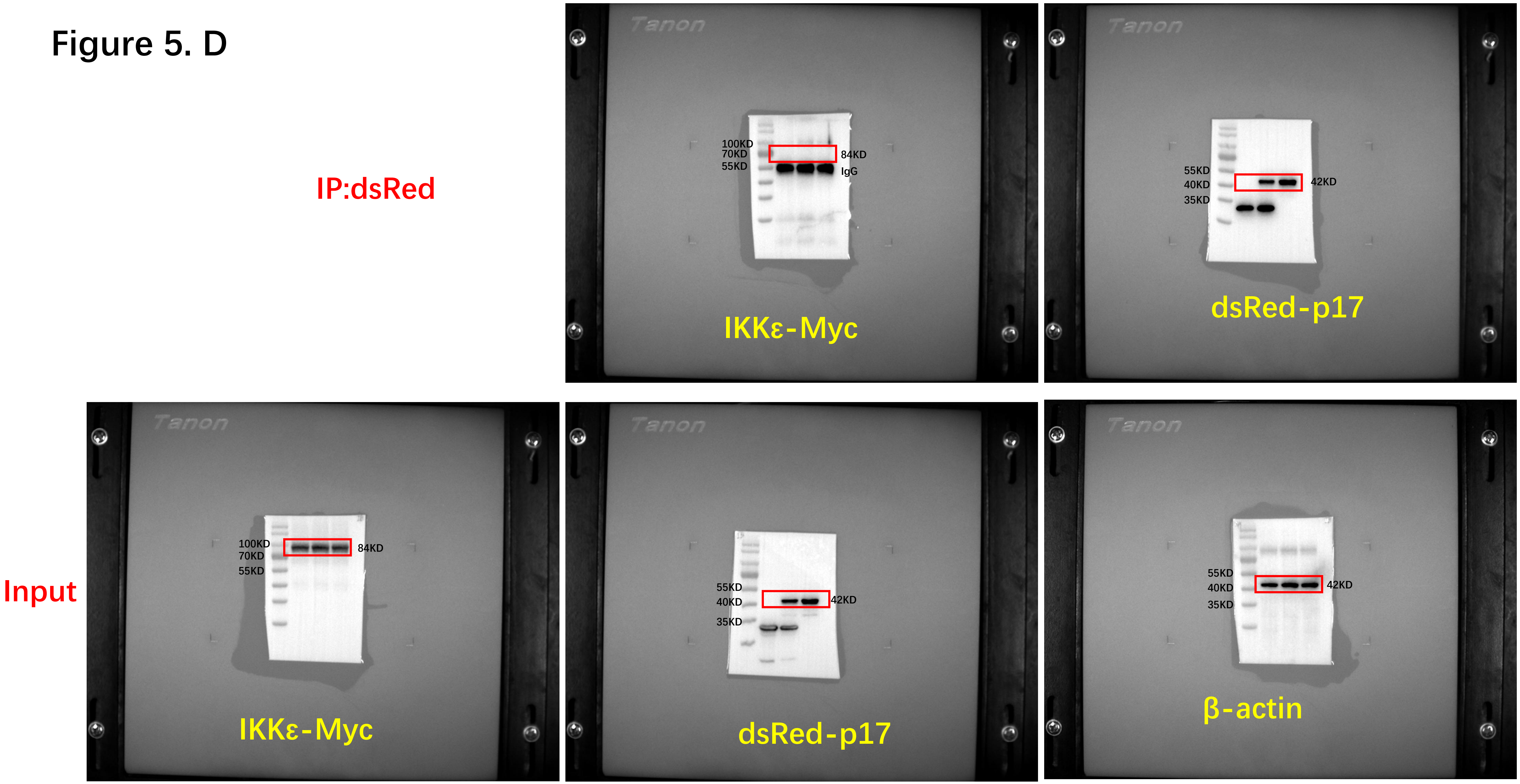
IP:dsRed
Input
100KD
70KD
55KD
84KD
IgG
IKKε-Myc
55KD
40KD
35KD
42KD
dsRed-p17
100KD
70KD
55KD
84KD
IKKε-Myc
55KD
40KD
35KD
42KD
dsRed-p17
55KD
40KD
35KD
42KD
β-actin

Figure 5. E

IP:GFP

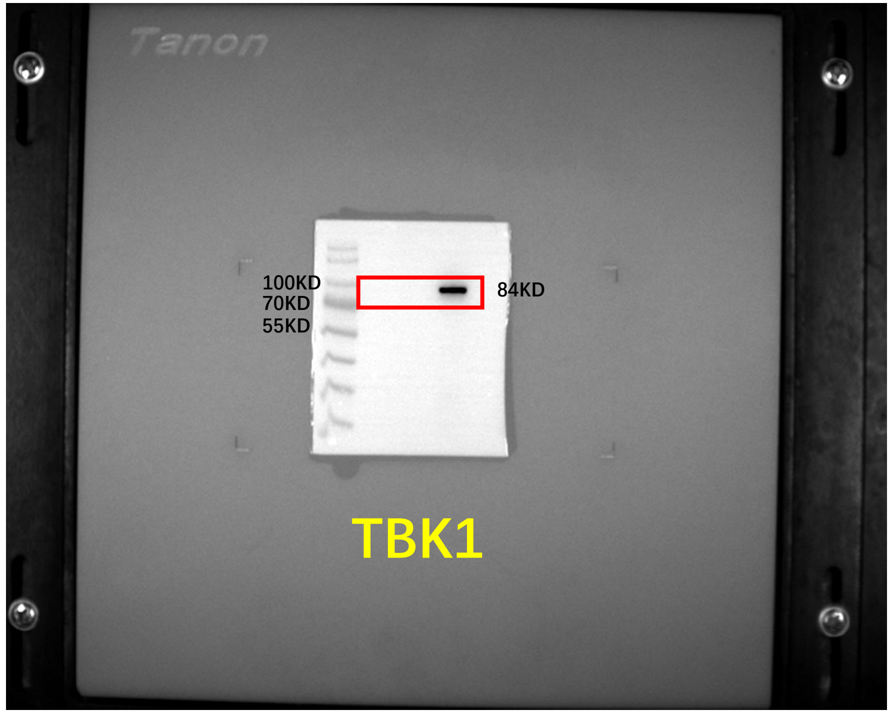

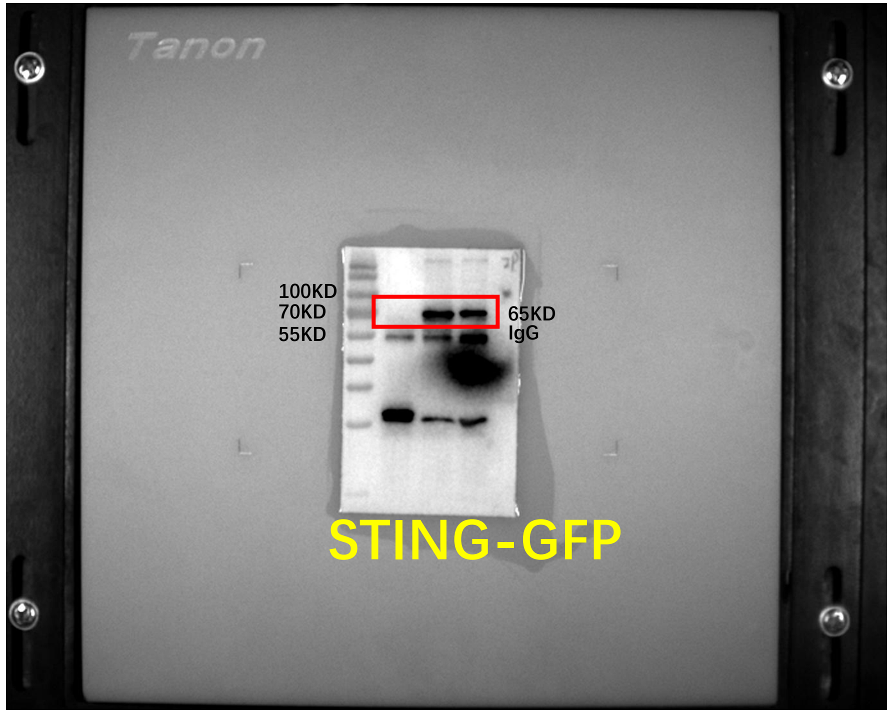

Figure 5. E

Input

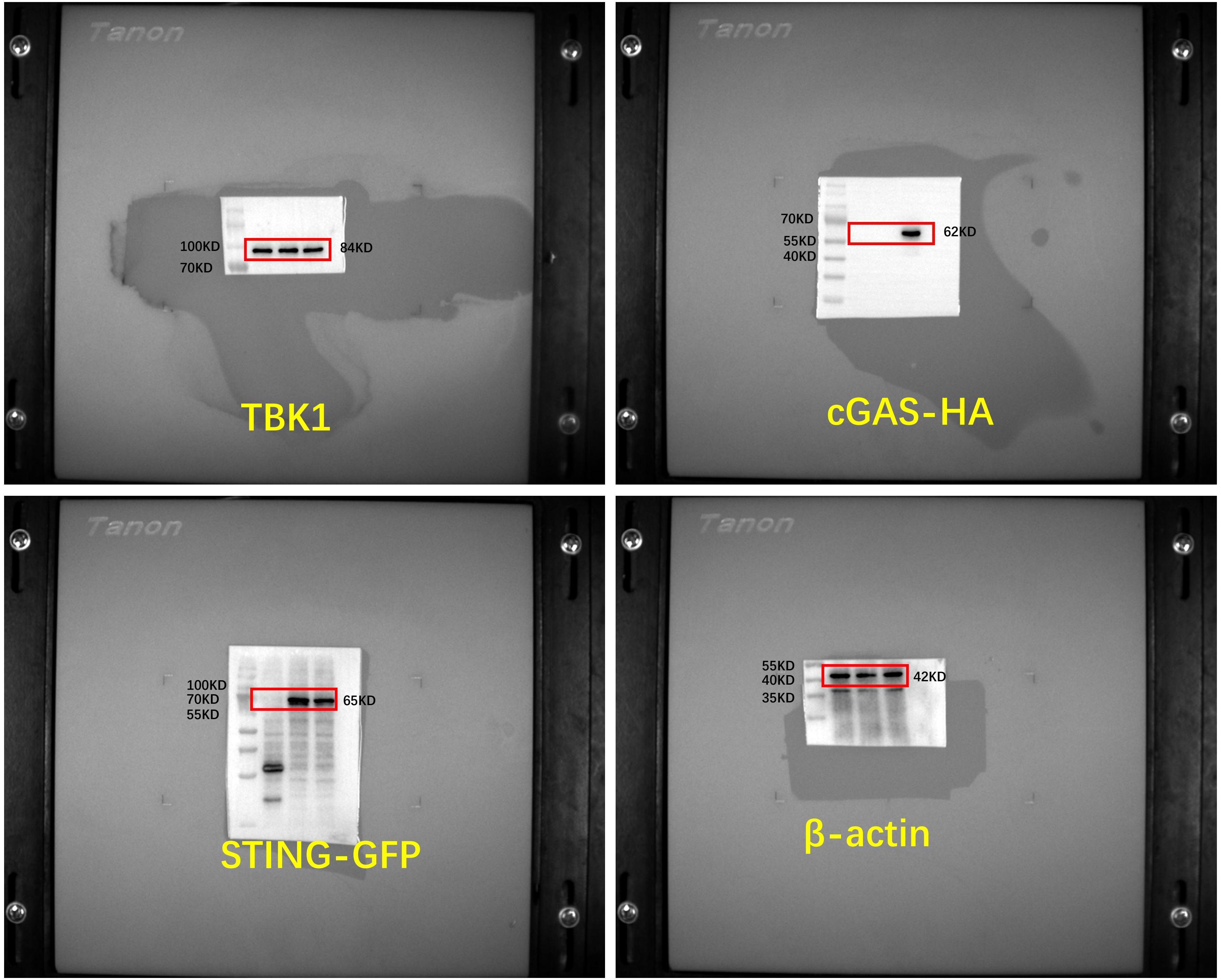

Figure 5. F

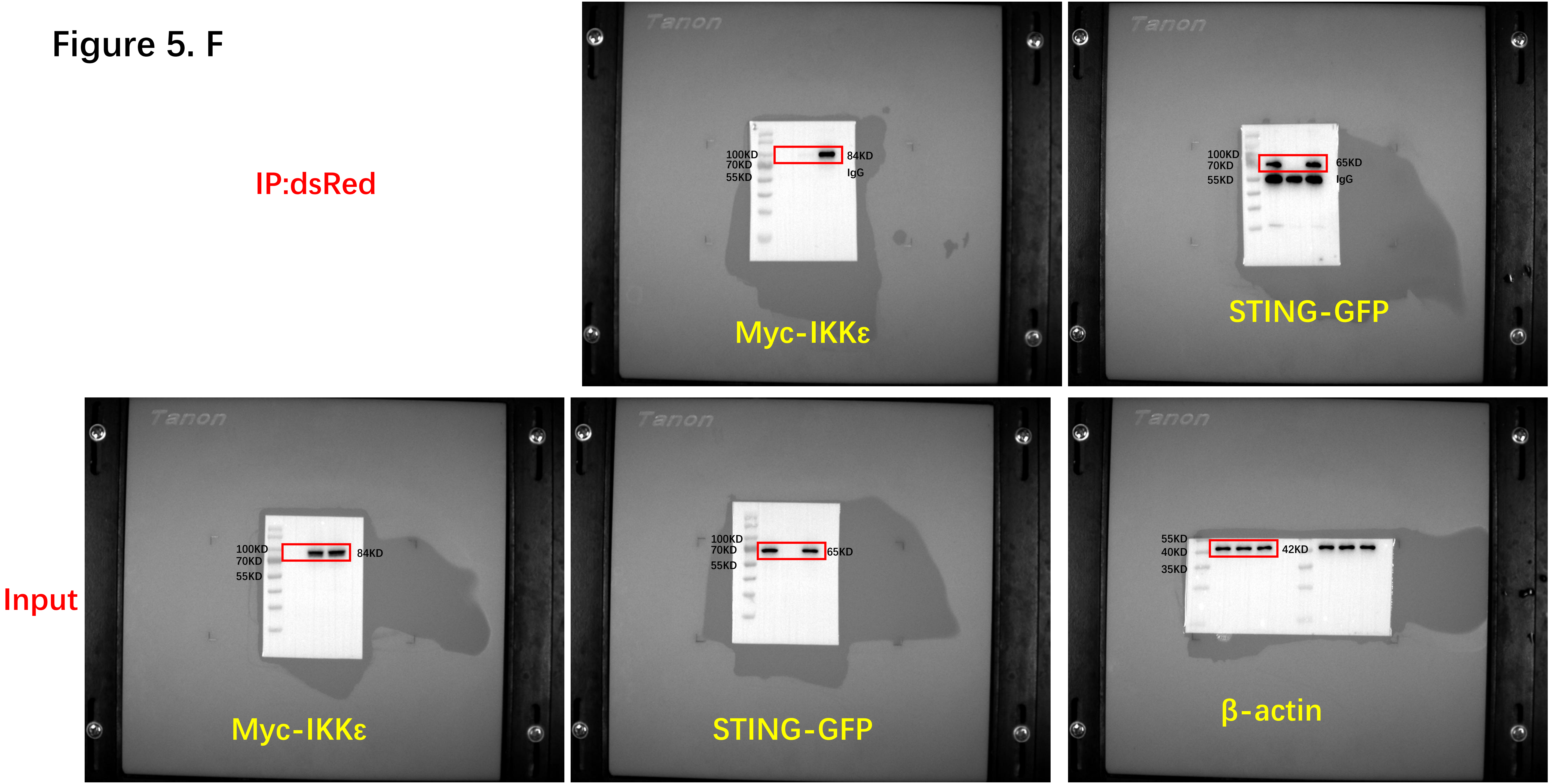
IP:dsRed
Input
100KD
70KD
55KD
84KD
IgG
Myc-IKKε
100KD
70KD
55KD
65KD
IgG
STING-GFP
100KD
70KD
55KD
84KD
Myc-IKKε
100KD
70KD
55KD
65KD
STING-GFP
55KD
40KD
35KD
42KD
β-actin

Figure 6. A

IP:GFP

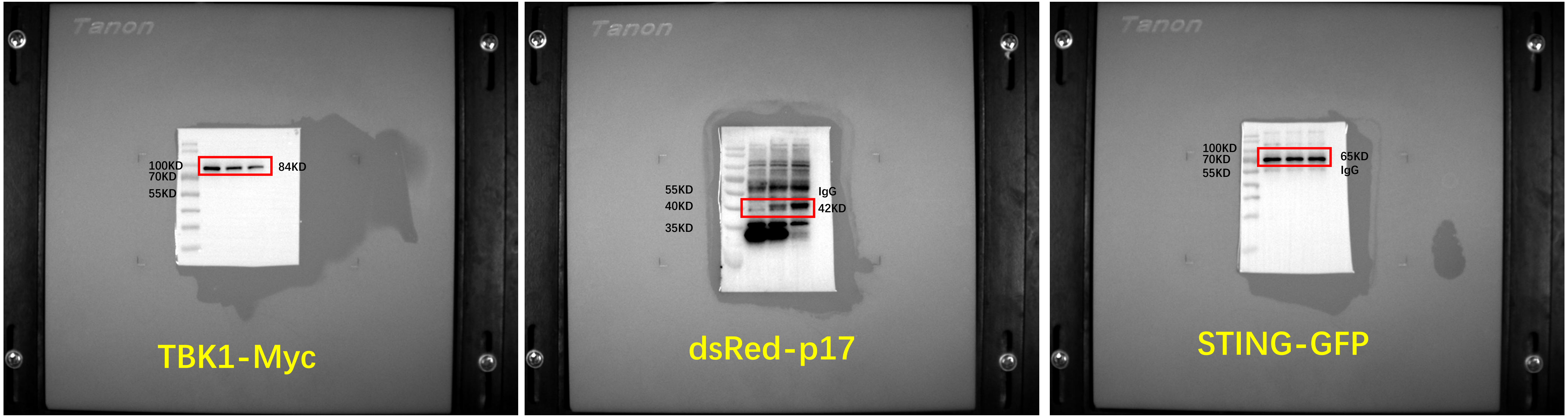

Figure 6. A

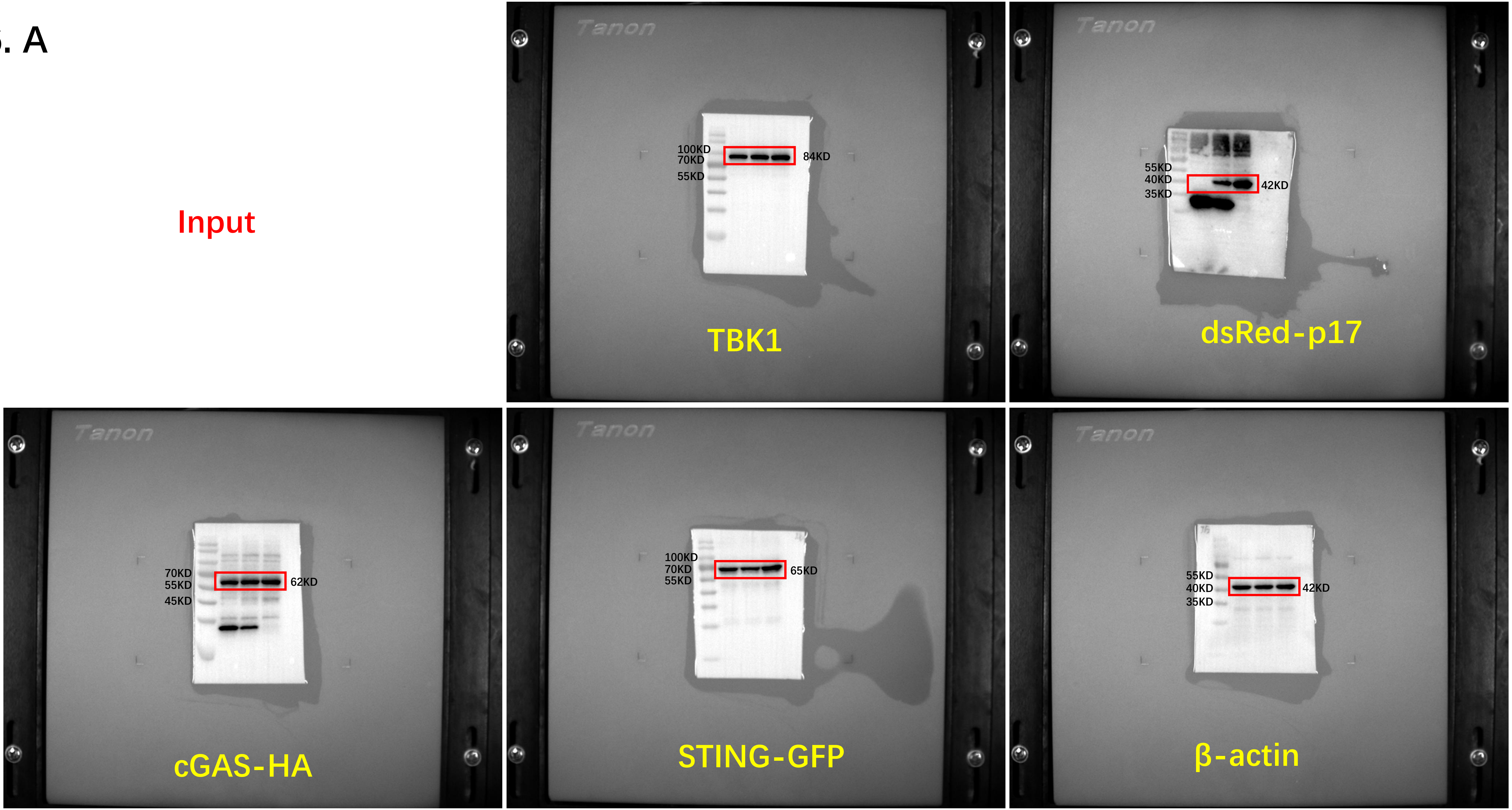

Input
100KD
70KD
55KD
84KD
TBK1
55KD
40KD
35KD
42KD
dsRed-p17
70KD
55KD
45KD
62KD
cGAS-HA
100KD
70KD
55KD
65KD
STING-GFP
55KD
40KD
35KD
42KD
β-actin

Figure 6. B

IP:GFP

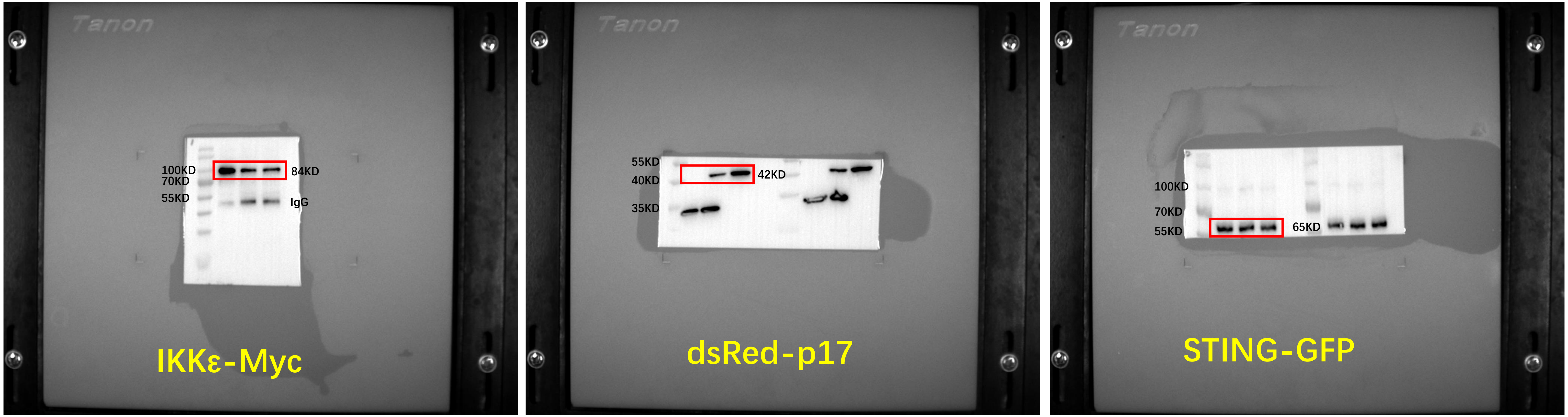

Figure 6. B

Input

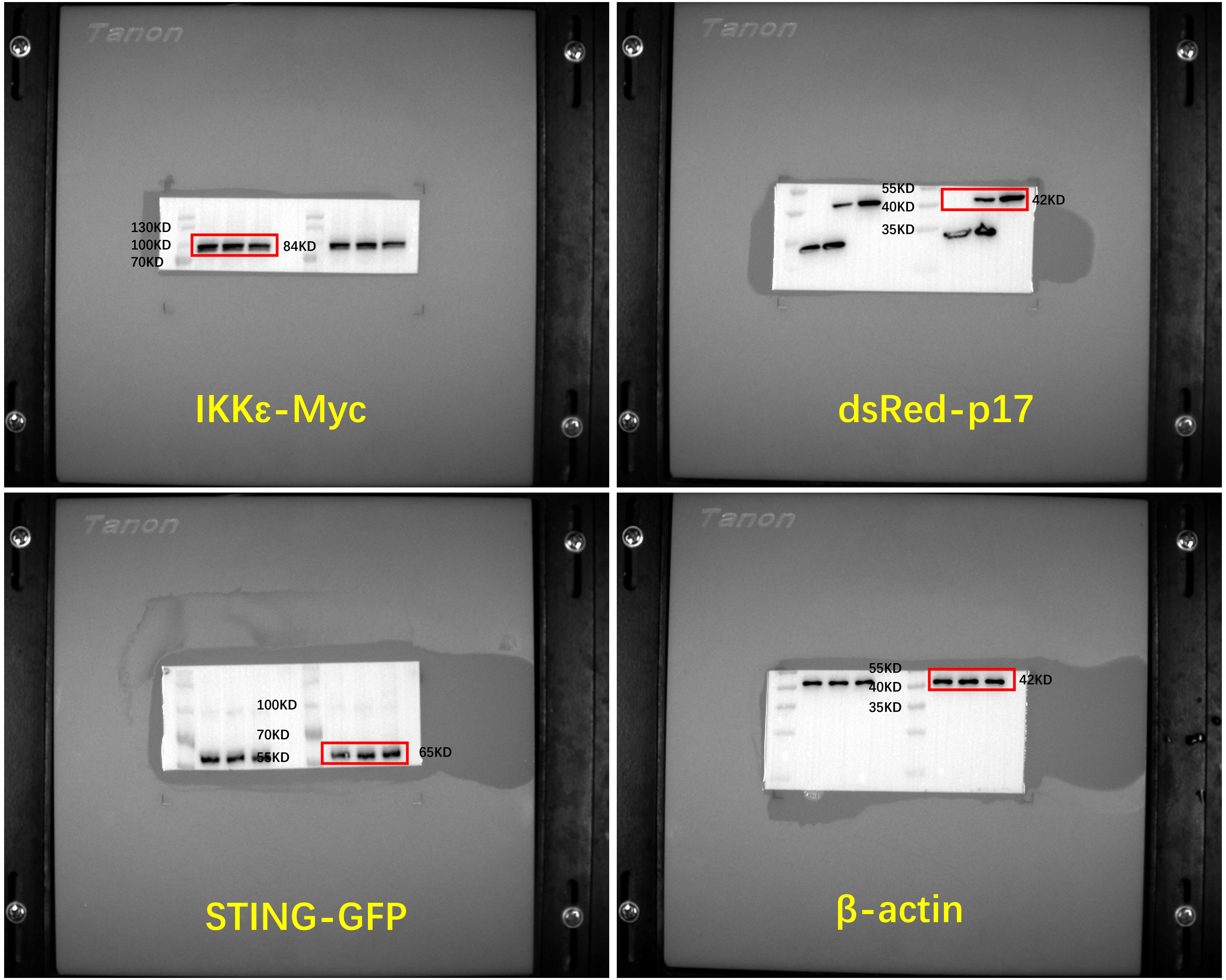

Figure 6. C

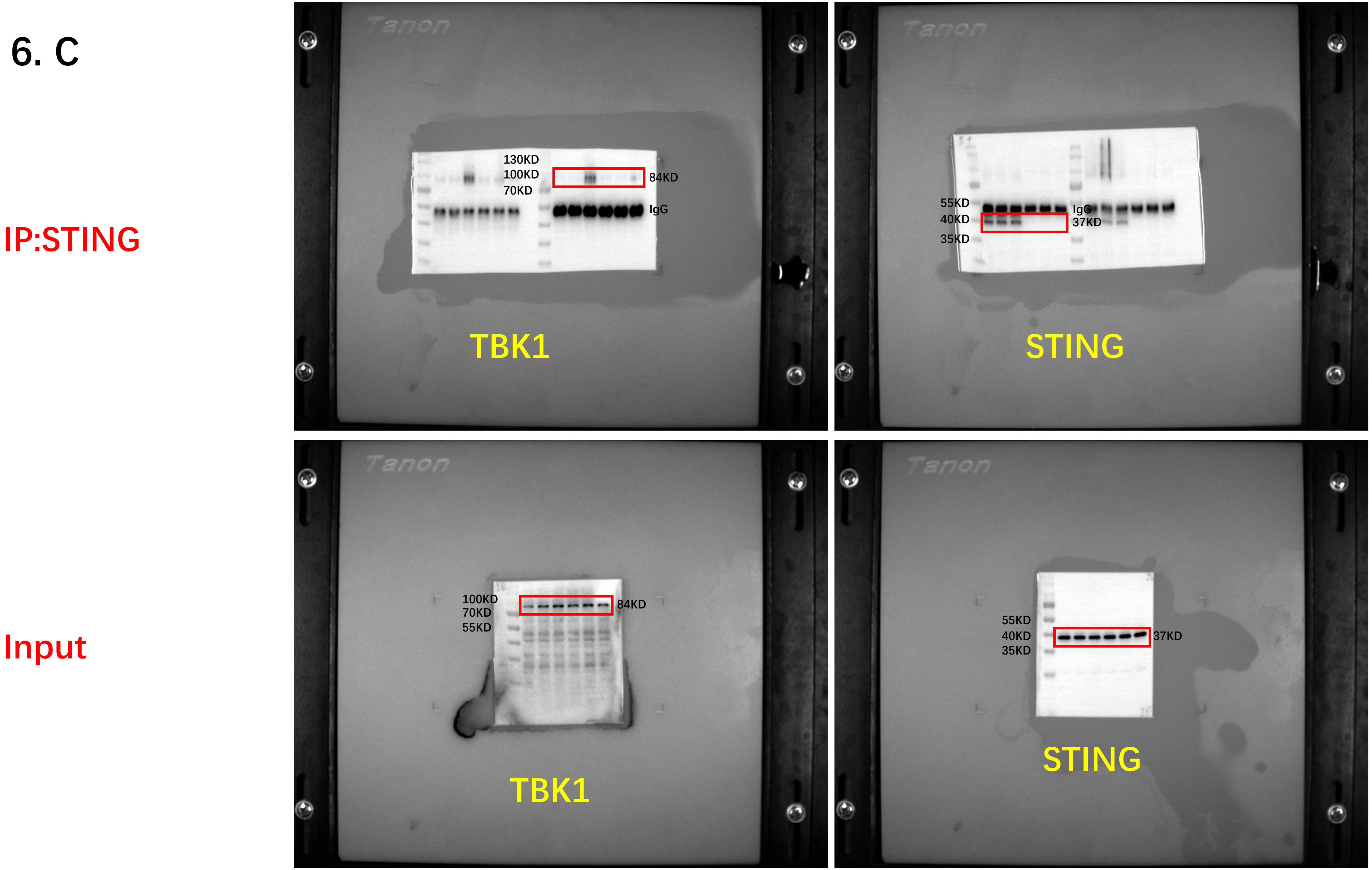
IP:STING
Input
130KD
100KD
70KD
84KD
IgG
TBK1
55KD
40KD
35KD
IgG
37KD
STING
100KD
70KD
55KD
84KD
TBK1
55KD
40KD
35KD
37KD
STING

Figure 6. D

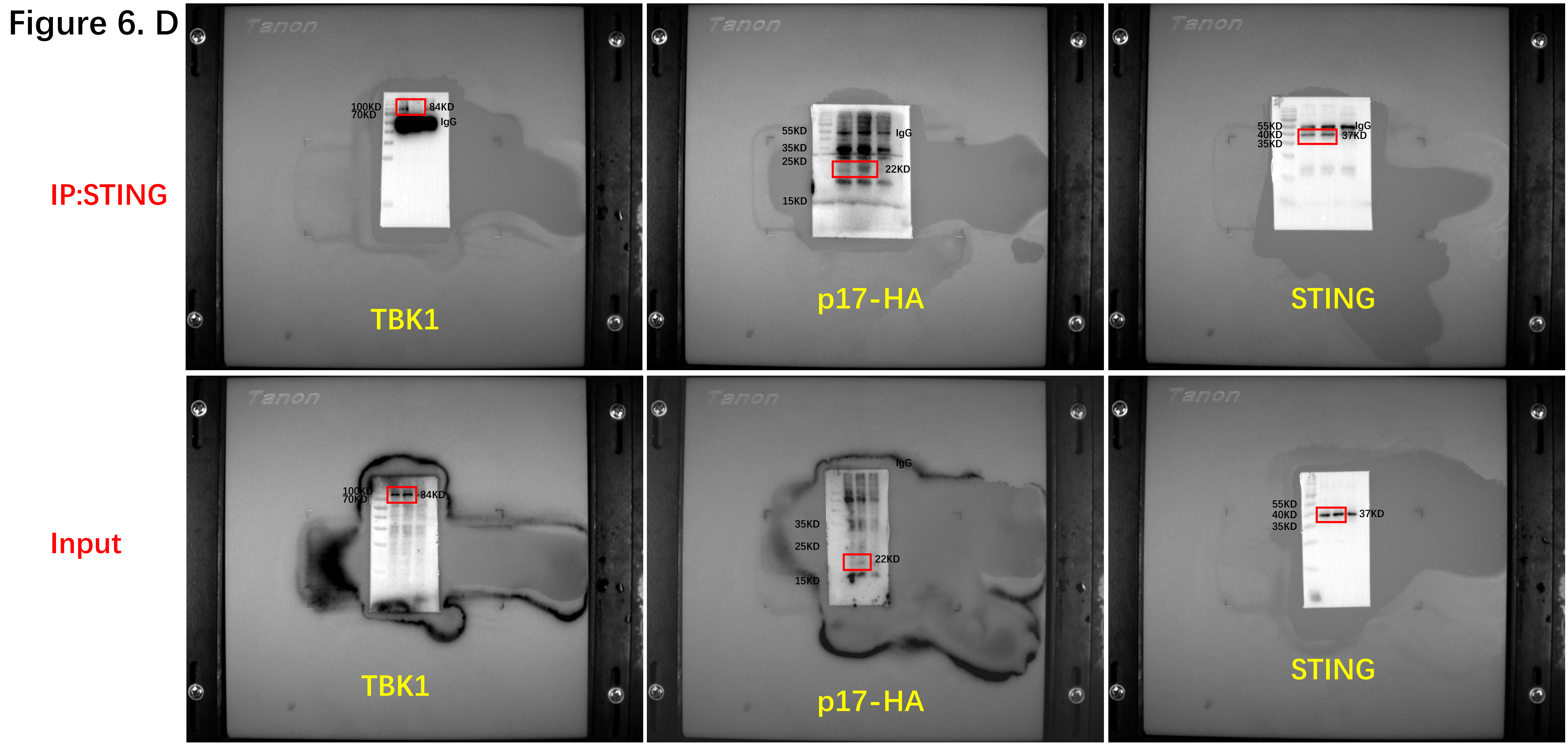

IP:STING
Input
100KD
70KD
84KD
IgG
TBK1
55KD
35KD
25KD
15KD
22KD
p17-HA
40KD
37KD
STING

Figure 8. A

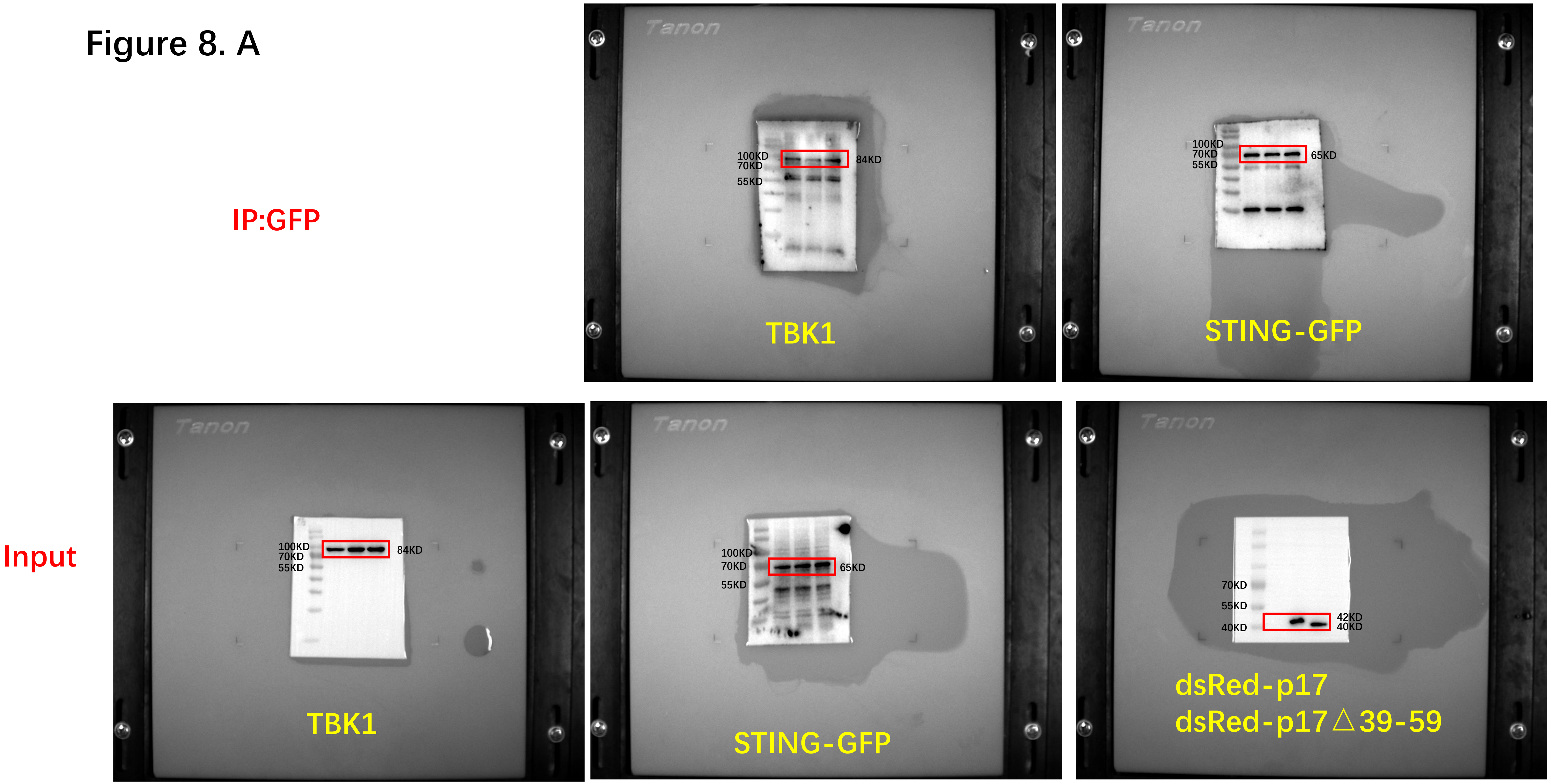
IP:GFP
Input
100KD
70KD
55KD
84KD
TBK1
100KD
70KD
55KD
65KD
STING-GFP
100KD
70KD
55KD
84KD
TBK1
100KD
70KD
55KD
65KD
STING-GFP
70KD
55KD
40KD
42KD
40KD
dsRed-p17
dsRed-p17△39-59

Figure 8. B

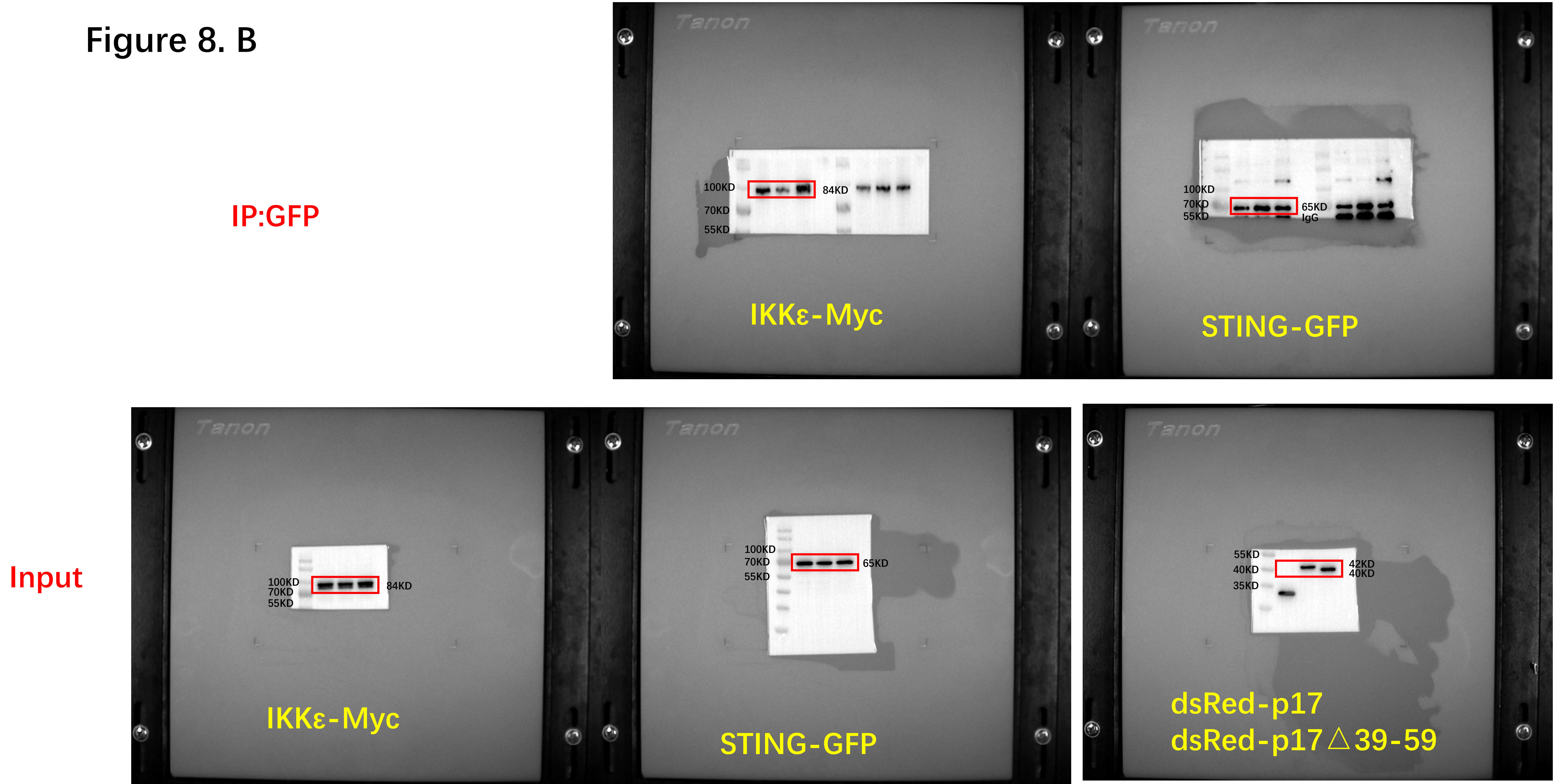

IP:GFP
100KD
70KD
55KD
84KD
IKKε-Myc
100KD
70KD
55KD
65KD
IgG
STING-GFP
Input
100KD
70KD
55KD
84KD
IKKε-Myc
100KD
70KD
55KD
65KD
STING-GFP
55KD
40KD
35KD
42KD
40KD
dsRed-p17
dsRed-p17△39-59

Figure 9. C

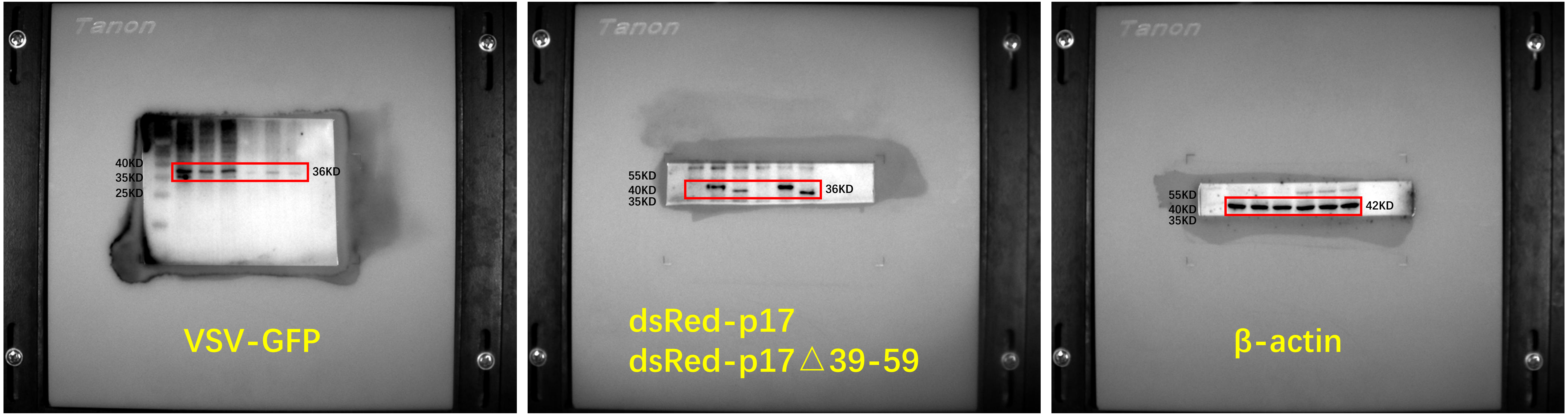
Tanon
40KD
35KD
25KD
36KD
VSV-GFP
Tanon
55KD
40KD
35KD
36KD
dsRed-p17
dsRed-p17△39-59
Tanon
55KD
40KD
35KD
42KD
β-actin

Figure 9. F

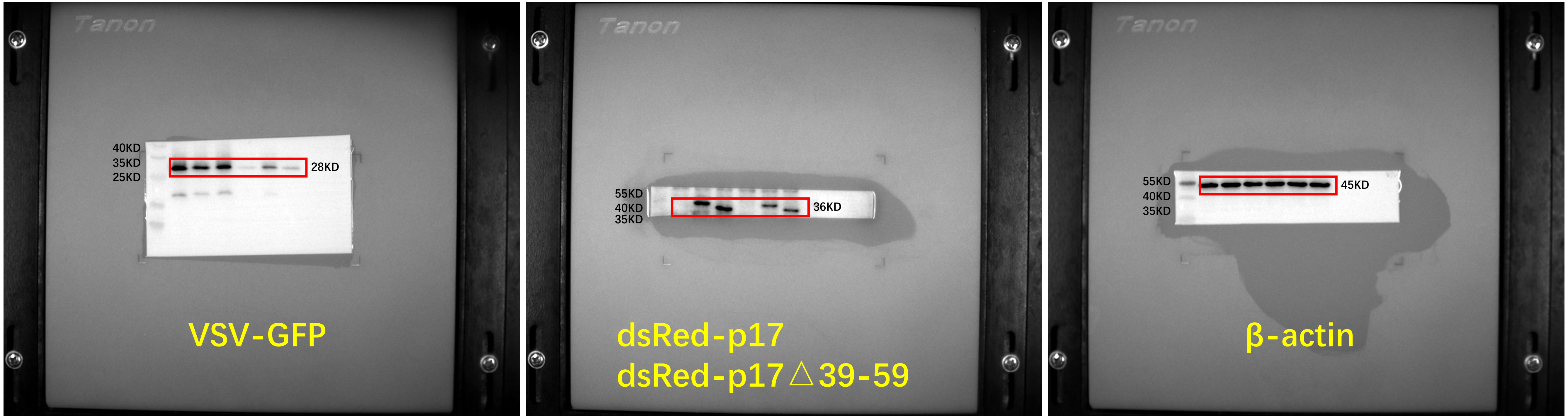
Tanon
40KD
35KD
25KD
28KD
VSV-GFP
Tanon
55KD
40KD
35KD
36KD
dsRed-p17
dsRed-p17△39-59
Tanon
55KD
40KD
35KD
45KD
β-actin

Figure 10 A

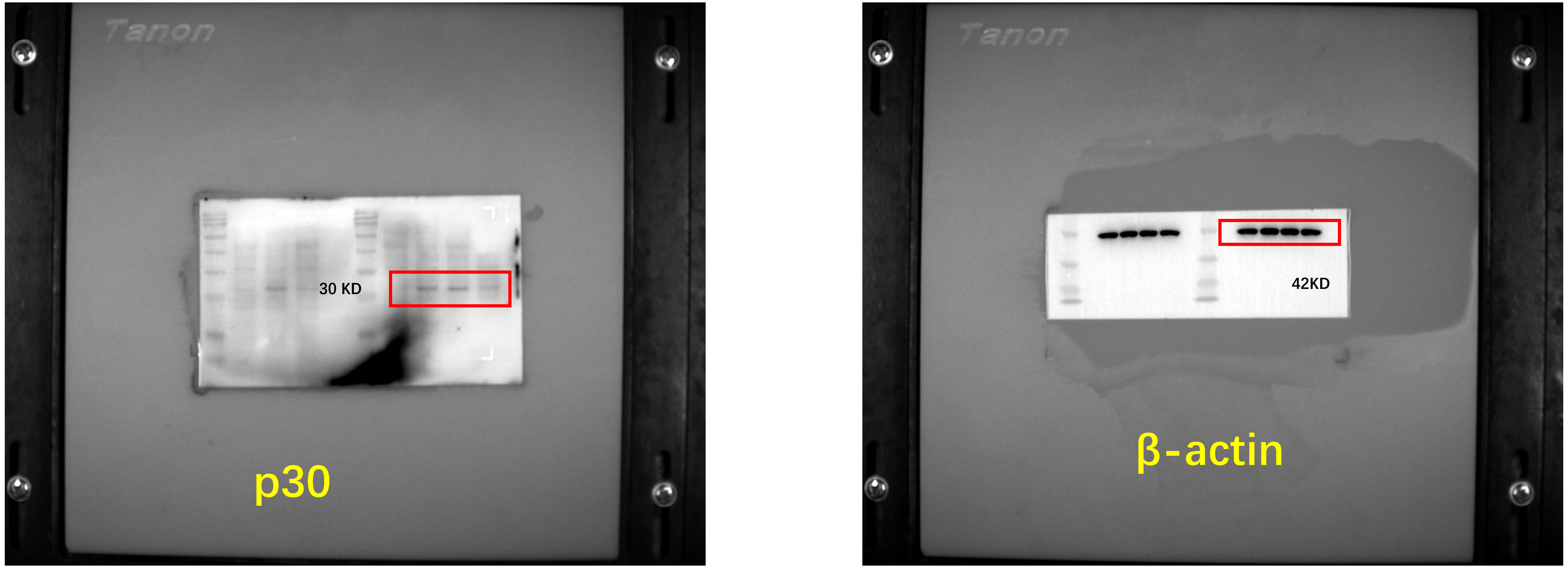

Supplement: Supplementary file 4 [file DataSheet_1.pdf]
