# Supplementary material for: African Swine Fever Virus Structural Protein p17 Inhibits cGAS-STING Signaling Pathway Through Interacting With STING
Source: Front Immunol. 2022 Jul 1;13:941579. doi: 10.3389/fimmu.2022.941579 (PMC9283692; doi:10.3389/fimmu.2022.941579)
Supplement: Supplementary file 5 [file Table_1.doc]

**Supplementary table 1. PCR Primers for p17 and pSTING cloning and mutations**

| **Primers** | **Sequence (5’-3’)** |
| --- | --- |
| **p17-2HA -F** | 5’-TGTCTCATCATTTTGGCAAAGAATTCATGGACACTGAAACGT-3’ |
| **p17-2HA -R** | 5’-ATCGTATGGGTAGCTGGTGATATCTGAATGCGCAAGTTCAGC-3’ |
| **pDsRed-C1-p17-F** | 5’-GCCGCCACCACCTGTTCCTGAGATCTATGGACACTGAAACGT-3’ |
| **pDsRed-C1-p17-R** | 5’-GGTACCGTCGACTGCAGAATTCTTATGAATGCGCAAGTTCAG-3’ |
| **pEGFP-N1-p17-F** | 5’-GTGAACCGTCAGATCCGCTAGCATGGACACTGAAACGTCTCC-3’ |
| **pEGFP-N1-p17-R** | 5’-GCTCACCATGGTGGCGACCGGTGGTGAATGCGCAAGTTCAGC-3’ |
| **p17 N12A-F** | 5’-CTCGCGGGTTGACAGGGCATGAGAAAGCAGTGGAGA-3’ |
| **p17 N12A-R** | 5’-TCTCCACTGCTTTCTCATGCCCTGTCAACCCGCGAG-3’ |
| **p17 N61A-F** | 5’-CAGTCAATAGTCCGGGCATAGTAAACGATGGCAACGATGATAAGAA-3’ |
| **p17 N61A-R** | 5’-TTCTTATCATCGTTGCCATCGTTTACTATGCCCGGACTATTGACTG-3’ |
| **p17 N97A-F** | 5’-GCAGGGAGGTGGAGGCTTTCCTTTTTCTAAAGAATACCGGGA-3’ |
| **p17 N97A-R** | 5’-TCCCGGTATTCTTTAGAAAAAGGAAAGCCTCCACCTCCCTGC-3’ |
| **p17 Δ39-43-F** | 5’-AATCGCCAAATATCCCGGAACAACTATTTTGATTTTGCTCATTATTATTCT-3’ |
| **p17 Δ39-43-R** | 5’-AGAATAATAATGAGCAAAATCAAAATAGTTGTTCCGGGATATTTGGCGATT-3’ |
| **p17 Δ44-48-F** | 5’-ACAACTGCGATTCTCCTGGGCATTATTATTCTTATCATCGTTG-3’ |
| **p17 Δ44-48-R** | 5’-CAACGATGATAAGAATAATAATGCCCAGGAGAATCGCAGTTGT-3’ |
| **p17 Δ49-53-F** | 5’-CTGGGCATTTTGATTTTGCTCATCGTTGCCATCGTTTACTAT-3’ |
| **p17 Δ49-53-R** | 5’-ATAGTAAACGATGGCAACGATGAGCAAAATCAAAATGCCCAG-3’ |
| **p17 Δ54-59-F** | 5’-GCATTTTGATTTTGCTCATTATTATTCTTATCTATAACCGGACTATTGACTGCAAG-3’ |
| **p17 Δ54-59-R** | 5’-CTTGCAGTCAATAGTCCGGTTATAGATAAGAATAATAATGAGCAAAATCAAAATGC-3’ |
| **p17 Δ39-48-F** | 5’-CGCCAAATATCCCGGAACAACTATTATTATTCTTATCATCGTTGC-3’ |
| **p17 Δ39-48-R** | 5’-GCAACGATGATAAGAATAATAATAGTTGTTCCGGGATATTTGGCG-3’ |
| **p17 Δ44-53-F** | 5’-TGCGATTCTCCTGGGCATCGTTGCCATCGTTT-3’ |
| **p17 Δ44-53-R** | 5’-AAACGATGGCAACGATGCCCAGGAGAATCGCA-3’ |
| **p17 Δ49-59-F** | 5’-CTGGGCATTTTGATTTTGCTCTATAACCGGACTATTGACTGC-3’ |
| **p17 Δ49-59-R** | 5’-GCAGTCAATAGTCCGGTTATAGAGCAAAATCAAAATGCCCAG-3’ |
| **p17 Δ39-59-F** | 5’-CAAATATCCCGGAACAACTAACCGGACTATTGACTGCA-3’ |
| **p17 Δ39-59-R** | 5’-TGCAGTCAATAGTCCGGTTAGTTGTTCCGGGATATTTG-3’ |
| **pSTING-F** | 5’-TTCGCAGATCTCCCTACTCCAGCCTGCATCCATCC-3’ |
| **pSTING-R** | 5’-TTCGCGAATTCTCAGAAGATATCTGAGCGGAGTGGAAGAGG-3’ |
| **pSTING-1-190- F** | 5’-TTCGCAGATCTCCCTACTCCAGCCTGCATCCATCC-3’ |
| **pSTING-1-190-R** | 5’-TTCGCGAATTCGAGTACGTTCTTGTGGCGCTGATTATAAGCTTGG-3’ |
| **pSTING-191-378-F** | 5’-TTCGCAGATCTGGGGGCATAGGGAACCACCGG-3’ |
| **pSTING-191-378-R** | 5’-TTCGCGAATTCTCAGAAGATATCTGAGCGGAGTGGAAGAGG-3’ |
| **pSTING-153-339-F** | 5’-TTCGCAGATCTTTCAACGTGGCTCATGGACTGGCC-3’ |
| **pSTING-153-339-R** | 5’-TTCGCGAATTCCTCCCTTTCCTCCTGCCGAAGG-3’ |
| **pSTING-1-338-F** | 5’-TTCGCAGATCTCCCTACTCCAGCCTGCATCCATCC-3’ |
| **pSTING-1-338-R** | 5’-TTCGCGAATTCCCTTTCCTCCTGCCGAAGGTGCC-3’ |
| **pSTING-339-378-F** | 5’-TTCGCAGATCTGAGGTTACCATGGGCAGTGCG-3’ |
| **pSTING-339-378-R** | 5’-TTCGCGAATTCTCAGAAGATATCTGAGCGGAGTGGAAGAGG-3’ |
| **pSTING-Δ238-241-F** | 5’-TGCTGGCATCAAGGGCAACAGCATCTATGAGC-3’ |
| **pSTING-Δ238-241-R** | 5’-GCTCATAGATGCTGTTGCCCTTGATGCCAGCA-3’ |
| **pSTING-Δ153-339 fragment 1-F** | 5’-TTCGCAGATCTCCCTACTCCAGCCTGCATCCATCC-3’ |
| **pSTING-Δ153-339 fragment 1-R** | 5’-CACTGCCCATGGTAACGTTCCTTTTTTCACA-3’ |
| **pSTING-Δ153-339 fragment 2-F** | 5’-TGTGAAAAAAGGAACGTTACCATGGGCAGTG-3’ |
| **pSTING-Δ153-339 fragment 2-R** | 5’-TTCGCGAATTCTCAGAAGATATCTGAGCGGAGTGGAAGAGG-3’ |

Note: the restriction enzyme sites are underlined.

**Supplementary table 2. Primers used for qPCR in this study**

| Primers | Sequences (5’-3’) |
| --- | --- |
| HSV1 gB gene -F | 5’-TTCTGCAGCTCGCACCAC-3’ |
| HSV1 gB gene -R | 5’-GGAGCGCATCAAGACCACC-3’ |
| VSV glycoprotein gene -F | 5’-TGCAAGGAAAGCATTGAACAA-3’ |
| VSV glycoprotein gene -R | 5’-GAGGAGTCACCTGGACAATCACT-3’ |
| Porcine IFN-β-F | 5’-TGAGCATTCTGCAGTACCTGA-3’ |
| Porcine IFN-β-R | 5’-CCGGAGGTAATCTGTAAGTCTGT-3’ |
| Porcine ISG15-F | 5’-ATCCTGGTGAGGAACGACAA-3’ |
| Porcine ISG15-R | 5’-GAAAGTCAGCCAGAACTGGTC-3’ |
| Porcine ISG56-F | 5’-ATGGGAGTTGGTCATTCAAGA-3’ |
| Porcine ISG56-R | 5’-CAGGTGTTTCACATAGGCCA-3’ |
| Porcine ISG60 F | 5’-CCAGACAACCCAGAATTCTCCT-3’ |
| PorcineISG60 R | 5’-AGAGCGCTGATGAAGTTGTTGC-3’ |
| Porcine IL-8-F | 5’-GTTTTTGAAGAGGGCTGAGAATTC-3’ |
| Porcine IL-8-R | 5’-CATGAAGTGTTGAAGTAGATTTGCTTG-3’ |
| Porcine β-actin-F | 5’-ATGAAGATCAAGATCATCGCG-3’ |
| Porcine β-actin-R | 5’-TCGTACTCCTGCTTGCTGATC-3’ |

**Supplementary table 3. The sequences of siRNA**

| siRNA Name | Primer Sequences (5′-3′) |
| --- | --- |
| p17 siRNA1-F | 5′-GCAAGUUCAGCUAAUUGUUTT-3′ |
| p17 siRNA1-R | 5′-AACAAUUAGCUGAACUUGCTT-3′ |
| p17 siRNA2-F | 5′-GCUCGACUUGCAGUCAAUATT-3′ |
| p17 siRNA2-R | 5′-UAUUGACUGCAAGUCGAGCTT-3′ |
| STING siRNA-F | 5′-CCAGCCUGCAUCCAUCCAUTT-3′ |
| STING siRNA-R | 5′-AUGGAUGGAUGCAGGCUGGTT-3′ |
| Control siRNA-F | 5′-UUCUCCGAACGUGUCACGUTT-3′ |
| Control siRNA-R | 5′-ACGUGACACGUUCGGAGAATT-3′ |
